# A randomized controlled trial of teprenone in terms of preventing worsening of COVID-19 infection

**DOI:** 10.1371/journal.pone.0287501

**Published:** 2023-10-26

**Authors:** Eiki Ichihara, Kou Hasegawa, Kenichiro Kudo, Yasushi Tanimoto, Kazuhiro Nouso, Naohiro Oda, Sho Mitsumune, Haruto Yamada, Ichiro Takata, Hideharu Hagiya, Toshiharu Mitsuhashi, Akihiko Taniguchi, Shinichi Toyooka, Kohei Tsukahara, Toshiyuki Aokage, Hirokazu Tsukahara, Katsuyuki Kiura, Yoshinobu Maeda

**Affiliations:** 1 Department of Allergy and Respiratory Medicine, Okayama University Hospital, Okayama, Japan; 2 Department of General Medicine, Okayama University Graduate School of Medicine, Dentistry, and Pharmaceutical Sciences, Okayama, Japan; 3 Department of Respiratory Medicine, National Hospital Organization Okayama Medical Center, Okayama, Japan; 4 Department of Allergy and Respiratory Medicine, National Hospital Organization Minami-Okayama Medical Center, Japan; 5 Department of Gastroenterology, Okayama City Hospital, Okayama, Japan; 6 Department of Internal Medicine, Fukuyama City Hospital, Fukuyama, Japan; 7 Department of Infectious Disease, Okayama City Hospital, Okayama, Japan; 8 Center for Innovative Clinical Medicine, Okayama University Hospital, Okayama, Japan; 9 Department of General Thoracic Surgery and Breast and Endocrine Surgery, Okayama University Graduate School of Medicine, Dentistry, and Pharmaceutical Sciences, Okayama, Japan; 10 Department of Emergency, Critical Care and Disaster Medicine, Okayama University Graduate School of Medicine, Dentistry and Pharmaceutical Sciences, Okayama, Japan; 11 Department of Pediatrics, Okayama University Graduate School of Medicine, Dentistry and Pharmaceutical Sciences, Okayama, Japan; 12 Department of Hematology, Oncology and Respiratory Medicine, Okayama University Graduate School of Medicine, Dentistry, and Pharmaceutical Sciences, Okayama, Japan; CHU Nantes, FRANCE

## Abstract

**Background:**

Some COVID-19 patients develop life-threatening disease accompanied by severe pneumonitis. Teprenone induces expression of heat-shock proteins (HSPs) that protect against interstitial pneumonia in preclinical models. We explored whether teprenone prevented worsening of COVID-19 infections.

**Methods:**

This open-label, randomized, pilot phase 2 clinical trial was conducted at five institutions in Japan. We randomized patients hospitalized for COVID-19 with fever to teprenone or no-teprenone groups in a 1:1 ratio. We stratified patients by sex, age < and ≥ 70 years and the existence (or not) of complications (hypertension, diabetes, ischemic heart disease, chronic pulmonary disease and active cancer). No limitation was imposed on other COVID-19 treatments. The primary endpoint was the intubation rate.

**Results:**

One hundred patients were included, 51 in the teprenone and 49 in the no- teprenone groups. The intubation rate did not differ significantly between the two groups: 9.8% (5/51) vs. 2.0% (1/49) (sub-hazard ratio [SHR] 4.99, 95% confidence interval [CI]: 0.59–42.1; p = 0.140). The rates of intra-hospital mortality and intensive care unit (ICU) admission did not differ significantly between the two groups: intra-hospital mortality 3.9% (2/51) vs. 4.1% (2/49) (hazard ratio [HR] 0.78, 95%CI: 0.11–5.62; p = 0.809); ICU admission 11.8% (6/51) vs. 6.1% (3/49) (SHR 1.99, 95%CI: 0.51–7.80; p = 0.325).

**Conclusion:**

Teprenone afforded no clinical benefit.

**Trial registration:**

Japan Registry of Clinical Trials jRCTs061200002 (registered on 20/May/2020).

## Introduction

Coronavirus disease 2019 (COVID-19) is caused by severe acute respiratory syndrome coronavirus 2 (SARS-CoV-2) and was first reported in Wuhan, China, in December 2019 [1]. Since that time, it has spread rapidly worldwide. Of all hospitalized COVID-19 patients, 26% required transfer to an intensive care unit (ICU) because of severe acute respiratory infection (SARI) [2]. Such patients exhibit elevated levels of tumor necrosis factor (TNF)-α and Th1/Th2 cytokines [3]; cytokine-induced cell damage is thought to aggravate infection. Certain immunosuppressive agents including dexamethasone and baricitinib improve the outcomes of patients with severe COVID-19 infections [4, 5]. However, the efficacy of cytoprotective agents has not been investigated.

Heat shock proteins (HSPs) are a family of proteins produced by cells in response to stressors such as heat, radiation, and infection. HSPs may play a role in COVID-19 infection [6]. COVID-19 invades cells via the angiotensin-converting enzyme 2 (ACE2) receptor [7]. HSPs are reported to modulate the effects of the renin-angiotensin-aldosterone (RAAS) system and thus may have a role in the infection of COVID-19. Teprenone exerts a cytoprotective effect by inducing HSPs, and is approved as a gastric mucosal protectant with a high safety profile [8]. In a mouse model of bleomycin-induced interstitial pneumonia, oral teprenone induced HSP70 in the lungs, reducing inflammatory cell infiltration and suppressing pulmonary fibrosis [9]. Teprenone suppressed pulmonary fibrosis in a C57BL/6 mouse model of radiation pneumonia by inducing HSP70 [10]. Thus, teprenone protects the lungs by inducing HSPs in various lung-injury models. Teprenone also induces thioredoxin (TRX), a stress-induced antioxidant enzyme with an–SH group that exhibits redox activity. TRX also exerts anti-inflammatory effects, including suppression of neutrophil/macrophage activities and complement activation [11]. TRX-1 reduced cytokine levels, neutrophil infiltration, and lung damage in a murine model of influenza pneumonia [12].

We hypothesized that teprenone might suppress exacerbation of COVID-19 pneumonia. We investigated whether teprenone inhibited worsening of COVID-19 patients.

## Patients and methods

### Trial design

This was an open-label, randomized, pilot phase 2 clinical trial conducted at five institutions in Japan. We randomized 100 patients hospitalized for COVID-19 with fever greater than or equal to 37.5°C to teprenone and no-teprenone groups in a 1:1 ratio using an online randomization system (the Internet Data and Information system for Clinical and Epidemiological research, a cloud version of the University Medical Information Network [UMIN]). This study was approved by the Certified Review Board of Okayama University (CRB20-001) and was registered at the Japan Registry of Clinical Trials (jRCTs061200002) on May 20, 2020. The study was conducted in compliance with the principles of the Declaration of Helsinki and informed consent was obtained from all patients before any screening procedure or inclusion. Participants were recruited from July 15, 2020, to August 18, 2021. Each patient was followed-up for 10 days or until discharge.

### Patients

All other treatments that attending physicians deemed necessary were allowed. The inclusion criteria were age ≥ 20 years at the time of enrolment, diagnosis of COVID-19 by polymerase chain reaction (PCR) or an antigen test, with fever defined as greater than or equal to 37.5°C, and provision of written informed consent. The threshold was 37.5°C because at that time the Japanese government recommended that anyone with a sustained fever of 37.5°C or higher seek medical attention. The exclusion criteria were: use of teprenone within 2 weeks prior to enrolment; a requirement for ventilation or extracorporeal membrane oxygenation; a concomitant active infection other than SARS-Co-2 (nontuberculous mycobacteriosis patients lacking progression for more than 1 month prior were excepted); lactating or pregnant women; and unidentified persons.

### Interventions

Patients in the teprenone group took 50 mg of teprenone orally three times a day for 10 days unless they developed intolerable toxicities. We imposed no limitation on other COVID-19 treatments in either group.

### Outcomes

The primary study endpoint was the intubation rate. The study protocol recommended that intubation be considered if either of the following criteria were met—PaO_2_ < 6 0mmHg under adequate oxygen supply, development of respiratory acidosis, decreased level of consciousness, or odd respiration. The secondary endpoints were the mortality and ICU admission rates; the times to fever decline defined as below 37.5°C for more than 24 hours without the use of antipyretics, supplementary oxygen termination and discharge, the maximum oxygen flow received by patients during hospitalization; and adverse events recorded in accordance with the fifth version of the Common Terminology Criteria for Adverse Events (CTCAE).

### Sample size

Assuming intubation rates of 10% in the teprenone group and 22% in the controls, the required sample size to afford a statistical power of 64% with a one-sided 0.1 type 1 error was 50 patients per arm. The intubation rate of 22% in controls was based on the weekly report (in Japanese) dated March 17, 2020, from the National Institute of Infectious Disease [13]. Of the 133 COVID-19 Japanese patients for whom intubation data were available, 29 required ventilation (22%).

### Statistical analyses

We used a minimization method for randomization. The stratification factors were (1) sex; (2) age less than 70 years; and, (3) one or more of the complications hypertension, diabetes, ischemic heart disease, chronic pulmonary disease, and active cancer. In this study, central randomization was used for allocation concealment.

Death was compared between groups using the Cox proportional hazard model. Sub-hazard ratios (SHRs) were calculated for intubation and admission to ICU using Fine and Gray’s method [14] because death was a competing event.

Considering that death was a competing risk, graphs were plotted using the cumulative incidence function with competing events for the analyses of time to oxygen termination and time to discharge. The Pepe and Mori test was used to compare groups [15], and SHRs were calculated using the Fine and Gray method. For the analysis of time to fever decline, death was not a competing risk because all the events of fever decline occurred before death. We used the log-rank test and Cox proportional hazard model to compare the teprenone and non-teprenone groups.

## Results

### Patients’ characteristics

**Fig 1:**
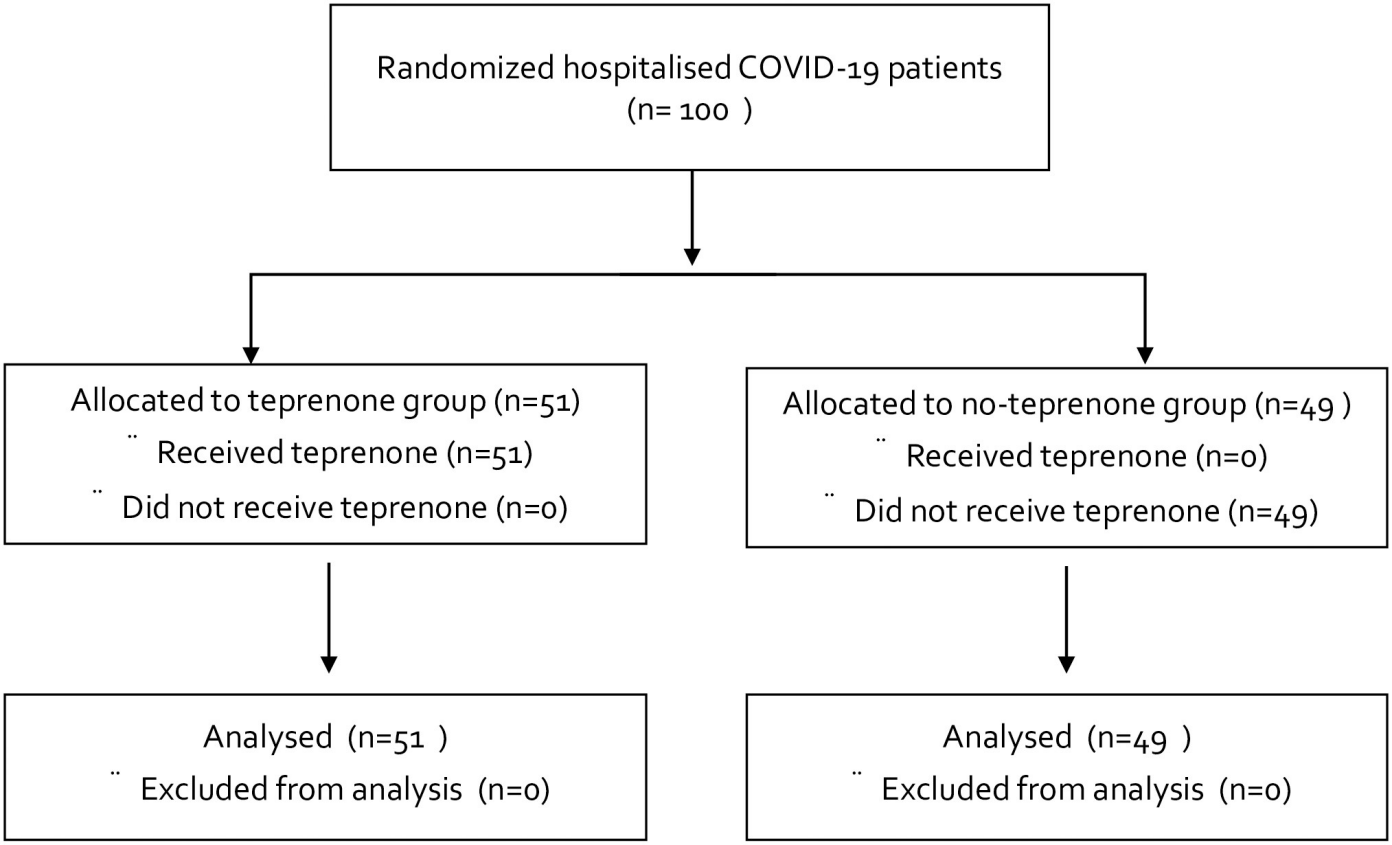
CONSORT flow diagram.

One hundred patients from five institutions were included (51 in the teprenone group and 49 in the non-teprenone group) between July 2020 and August 2021 (Fig 1). In this period, the SARS-CoV-2 Alpha and Delta variants were dominant in Japan. Patient characteristics are listed in Table 1. The groups were well-balanced in terms of the median time from disease onset to randomization: 7 vs. 5 days; age: 63 vs. 64 years; body mass index: 23.6 vs. 24.2 kg/m^2^; female sex: 41 vs. 39%; and smoking status: 49 vs, 49%. The frequencies of co-existing conditions (hypertension, diabetes, cardiovascular disease, chronic pulmonary disease, and active cancer) were also similar. Of the teprenone group 55% had a World Health Organization Ordinary Score of 3, as did 63% of the non-teprenone group. More than half the patients in each group did not require oxygen at baseline (53% of the teprenone group and 63% of the non-teprenone group). The white blood cell, lymphocyte, creatinine, and CRP levels were similar in the two groups.

**Table 1:** Patients’ characteristics.

	teprenone (n = 51)	No teprenone (n = 49)
Time from onset to randomization (days)		
Median (range)	7 (2–15)	5 (2–15)
Age, years		
Median (range)	63 (21–88)	64 (22–86)
Body mass index		
Median (range)	23.6 (15.2–37.2)	24.2 (16.5–42)
Sex		
Female	21 (41%)	19 (39%)
Male	30 (59%)	30 (61%)
Smoking history		
Yes	25 (49%)	24 (49%)
No	20 (39%)	23 (47%)
Unknown	6 (12%)	2 (4%)
Coexisting conditions		
Hypertension	19 (37%)	16 (32%)
Diabetes	10 (20%)	10 (20%)
Cardiovascular disease	4 (8%)	4 (8%)
Chronic pulmonary disease	3 (6%)	4 (8%)
Malignant neoplasm	1 (2%)	1 (2%)
WHO Ordinary Score for Clinical Investigation		
3	28 (55%)	31 (63%)
4	23 (45%)	17 (35%)
5	0 (0%)	1 (2%)
Oxygen administration at registration		
Yes	24 (47%)	18 (37%)
No	27 (53%)	31 (63%)
Laboratory values, median (range)		
WBC count, /μL	4,620 (1,800–12,720)	5,220 (2,500–11,500)
Lymphocytes, /μL	1,020 (151–1930)	1,040 (60–4,850)
Creatinine, mg/dL	0.80 (0.49–12.17)	0.81 (0.50–14.13)
CRP, mg/dL	2.14 (0.01–19.95)	1.56 (0.03–19.70)

WHO: World Health Organization, WBC: white blood cell

### Treatments

Among the 51 teprenone group patients, 43 (84%) took teprenone as prescribed for 10 days, and 5 (10%) took teprenone for 8 or 9 days. The remaining three (6%) took teprenone for less than 6 days because of their severe state or refusal.

Treatments other than teprenone for COVID-19 are listed in Table 2. Systemic steroid was administered in 71% of the teprenone group and 61% of the non-teprenone group; remdesivir was administered in 47% and 47%, respectively. Tocilizumab was used in 16% of the teprenone group and 4% of the non-teprenone group.

**Table 2:** Treatments other than teprenone for COVID-19.

	teprenone (n = 51)	No teprenone (n = 49)
Steroid	36 (71%)	30 (61%)
Dexamethasone	35 (69%)	29 (59%)
mPSL (pulsed)	6 (12%)	8 (16%)
Remdesivir	24 (47%)	23 (47%)
Tocilizumab	8 (16%)	2 (4%)

mPSL: methylprednisolone

### Outcomes

**Fig 2:**
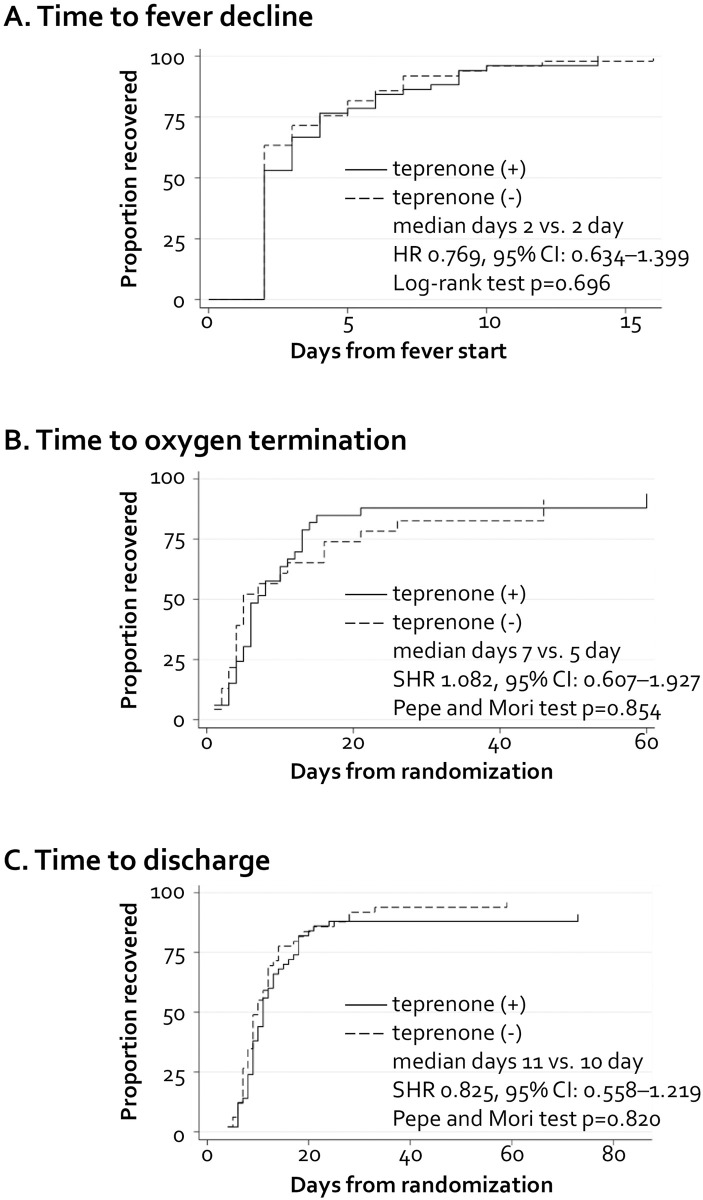
Times to clinical improvement. A. Times to fever decline. B. Times to supplementary oxygen termination. C. Times to discharge.

**Fig 3:**
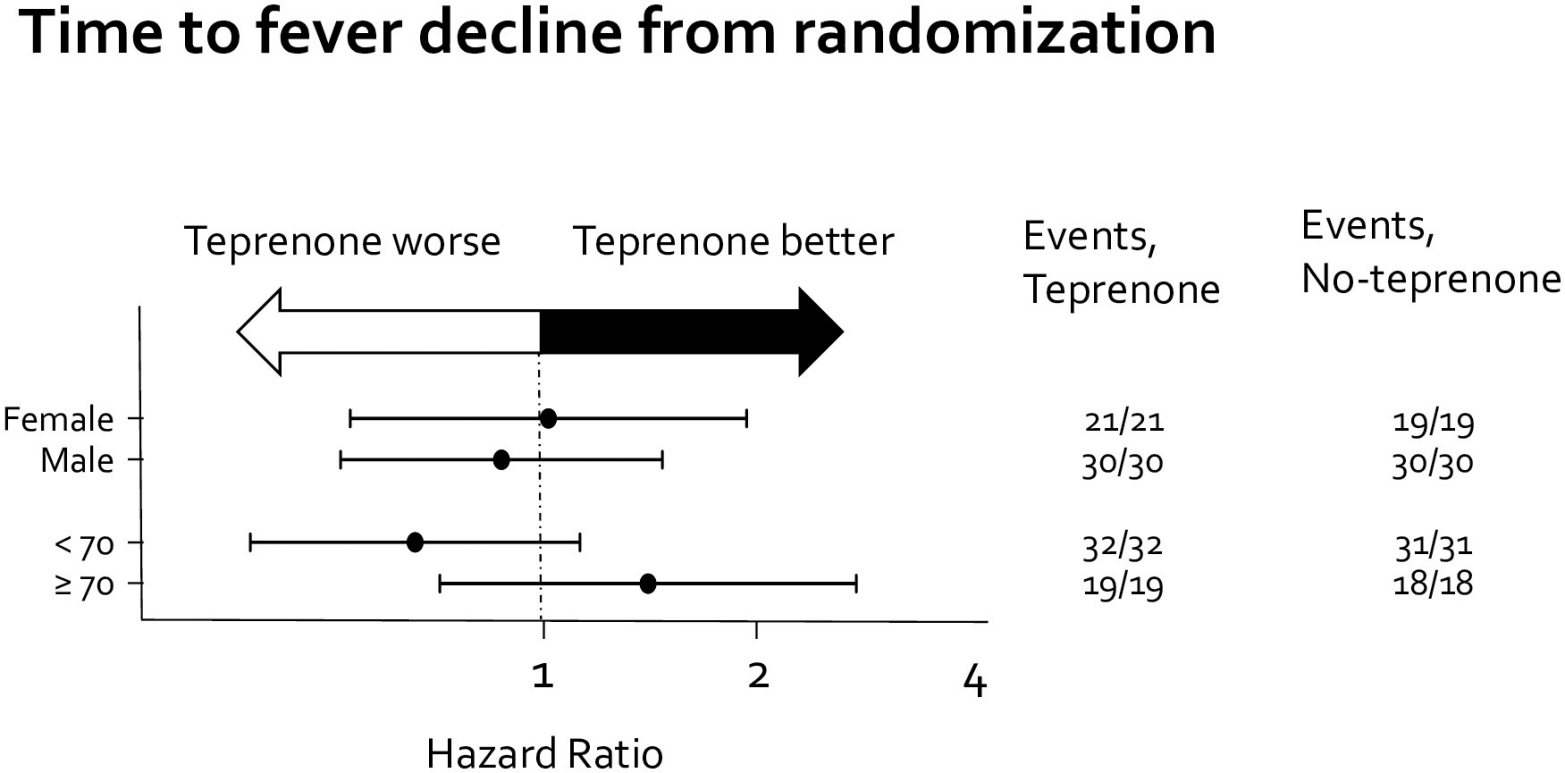
Forest plot of the times to fever decline.

There were no significant differences in the rates of intra-hospital mortality, intubation, or ICU admission (Table 3). For intra-hospital mortality, the hazard ratio (HR) was 0.78 (95% CI: 0.11–5.62; p = 0.809); for intubation, the SHR was 4.99 (95% CI: 0.59–42.1; p = 0.140); and for ICU admission, the SHR was 1.99 (95% CI: 0.51–7.80; p = 0.325). The rate of need for oxygen inhalation of more than 5 L/min did not differ between the groups (Table 4; 15.6 vs. 18.3%, p = 0.79). Furthermore, there were no significant between-group differences in median time to fever decline, 2 vs. 2 days (HR: 0.769, 95% CI: 0.634–1.399, p = 0.769; log-rank test, p = 0.696) (Fig 2A); median time to oxygen termination, 7 vs. 5 days (SHR: 1.082, 95% CI: 0.607–1.927, p = 0.788; Pepe and Mori test p = 0.854, p for competing event = 0.355) (Fig 2B); or median time to discharge, 11 vs. 10 days (SHR: 0.825, 95% CI: 0.558–1.219, p = 0.335; Pepe and Mori test p = 0.820, p for competing event = 0.638) (Fig 2C). Finally, we performed a subgroup analysis by time to fever decline (Fig 3). In subgroups divided by sex and age, we found no significant difference in fever duration.

**Table 3:** Severity rate.

	teprenone	No teprenone	HR/SHR
	n = 51	n = 49	
Intra-hospital mortality			
% (n)	3.9% (2)	4.1% (2)	0.78
95%CI%	4.8–13.5%	5.0–14.0%	0.11–5.62
			p = 0.809
Intubation			
% (n)	9.8% (5)	2.0% (1)	4.99
95%CI	3.2–21.4%	0.1–10.9%	0.59–42.1
			p = 0.140
Admission to ICU			
% (n)	11.8% (6)	6.1% (3)	1.99
95%CI	4.4–23.9%	1.3–16.9%	0.51–7.80
			p = 0.325

CI: confidence interval, ICU: intensive care unit, HR: hazard ratio, SHR: sub-hazard ratio.

HRs were calculated for intra-hospital mortality because there were no competing events. For the other failure events, sub-hazard ratios were calculated with death as a competing event.

**Table 4:** Maximum oxygen demand.

	Teprenone	No teprenone	
	n = 51	n = 49	
Maximum oxygen demand more than 5L/min			
% (n)	15.6% (8)	18.3% (9)	p = 0.79
95%CI	7.0–28.5%	8.7–32.0%	

CI: confidence interval

### Safety

Of the 51 patients given teprenone, none developed intolerable toxicities with teprenone. Two experienced adverse effects that we considered attributable to teprenone. One was diarrhea (CTCAE grade 2) and the other was liver enzyme elevation (also grade 2), neither of which led to teprenone discontinuation.

## Discussion

We performed an open-label, multi-center, randomized clinical trial to explore whether teprenone induced HSPs that prevented worsening of COVID-19. Unfortunately, teprenone was not effective.

In a retrospective analysis, the survival rate was significantly higher in severe COVID-19 patients who received, as compared to those who did not receive, Jusvinza, a derivative of HSP60, (90.4 vs. 39.5%, p < 0.0001). Also, inflammation and coagulation biomarkers were significantly reduced in the Jusyinza group [16]. This implies that HSPs regulate inflammation in COVID-19 patients, thus improving their outcomes [5, 17, 18]. In this study, we explored whether teprenone, an inducer of HSPs, protected cells against cytokine storm in COVID-19 patients. Teprenone has been used widely in Japan for more than 20 years and exhibits few adverse effects [8].

In this study, the patients in the teprenone group were registered later (time from disease onset to randomization 7 vs. 5 days), which implies less severe infection since they did not need intubation until at least 7 days after onset, However, despite such a potentially favorable factor, teprenone did not show a benefit in this study.

There are several potential reasons why teprenone lacked clinical activity. First, the intubation rate (the primary endpoint) was lower than expected. Based on initial cohort data, we had assumed that the rate would be 22% in the no-teprenone group, whereas the actual rate was 2% (Table 3); thus, this study was underpowered. Second, teprenone may be effective in some patients, but not in those who participated in this study. Heat shock response (HSR) is downregulated in critically ill COVID-19 patients [19], whereas most of the patients in this study did not have severe COVID-19 and more than half were not on oxygen at registration. Third, we used teprenone at the dose employed for gastric mucosal protection, which may be too low to induce HSPs in the lung. We will determine serum HSP levels in a future study. Finally, teprenone may have no activity against COVID-19. There have been some reports of the efficacy of HSPs against COVID-19 [6, 16], but other findings indicate a pro-viral effect [20–22]. To determine the role of HSPs in COVID-19 infection, further research is needed.

Our study had certain limitations. First, this was an open-label non-blinded study; bias was possible. This study started in the early stages of the COVID-19 pandemic when many facilities were not accustomed to handling COVID-19 inpatients. We considered that blinding and a placebo would complicate the study and increase the risk of infection for medical staff. Therefore, blinding was not implemented in this study. Second, this was a phase 2 study with a small number of patients, so the results are not definitive. The study has a low power of 64% at a one-sided 0.1% significance level. This is because this study was conducted in the early stages of the COVID-19 pandemic when it was difficult to estimate the number of COVID-19 cases; we thought that the setting would be achievable or realistic. Third, the decision to intubate is subjective and may have been affected by factors other than disease severity. Furthermore, the intubation rate was lower than estimated, limiting the generalizability of the results. Therefore, the findings should be interpreted with caution. Finally, this study was conducted entirely in Japan, so the generalizability of our results to other countries remains unclear.

## Conclusions

Teprenone was of no clinical benefit.

## Supporting information

S1 Checklist CONSORT checklist. (DOC)

S1 Protocol Translation of the protocol. (DOCX)

S2 Protocol Original protocol. (DOCX)

S1 Data Clinical data of patients. (XLSX)

S1 File (DOCX)

## Abbreviations

COVID-19: Coronavirus disease 2019
HSP: Heat-shock protein
ICU: Intensive care unit
PCR: Polymerase chain reaction
ROS: Reactive oxygen species
SARI: Severe acute respiratory infection
SARS-CoV-2: Severe acute respiratory syndrome coronavirus 2
TNF: Tumor necrosis factor
TRX: Thioredoxin
UMIN: University Medical Information Network
