# Supplementary material for: A randomized controlled trial of teprenone in terms of preventing worsening of COVID-19 infection
Source: PLoS One. 2023 Oct 26;18(10):e0287501. doi: 10.1371/journal.pone.0287501 (PMC10602324; doi:10.1371/journal.pone.0287501)
Supplement: S1 Protocol — (DOCX) [file pone.0287501.s002.docx]

A randomised controlled trial of teprenone in terms of preventing worsening of COVID-19 infections

Study Protocol (original protocol in Japanese)

【Principal Investigator】

Eiki Ichihara

　Department of Allergy and Respiratory Medicine, Okayama University Hospital

【Planned research period】

　From the date of jRCT publication to Dec. 31. 2024.

Table of Contents

[１．Study title 4](#_Toc95570398)

[２．Organization of the research 4](#_Toc95570399)

[３．Background, objective and significance of the study 4](#_Toc95570400)

[４．Methods 5](#_Toc95570401)

[５．Observation and test items 7](#_Toc95570402)

[６．Evaluation Items 9](#_Toc95570403)

[７．Target number of the included patients and rationale for setting 9](#_Toc95570404)

[８．Data aggregation and statistical analysis methods 9](#_Toc95570405)

[９．Research period 10](#_Toc95570406)

[１０．Inclusion and exclusion criteria 10](#_Toc95570407)

[１１．Background and scientific rationale for the study (rationale and validity of the study) 11](#_Toc95570408)

[１２．Procedures for obtaining informed consent 11](#_Toc95570409)

[１３．Handling of personal information, etc. (including methods of anonymization) 12](#_Toc95570410)

[１４．Burdens, anticipated risks, and benefits to research subjects, a comprehensive assessment of these, and measures to minimize such burdens and risks 12](#_Toc95570411)

[１５．Method of storage and destruction of information including storage period 13](#_Toc95570412)

[１６．Matters related to access to source documents, etc. 14](#_Toc95570413)

[１７．Periodic reporting 14](#_Toc95570414)

[１８．Status of conflicts of interest related to research and other research, including sources of funding for research, conflicts of interest related to research for the duration of the research, and personal earnings 15](#_Toc95570415)

[１９．Methods of disclosing information about research (registration of research plans and publication of research results) 15](#_Toc95570416)

[２０．Response to consultation, etc. from research subjects, etc. 15](#_Toc95570417)

[２１．Procedures for obtaining informed consent from a surrogate, etc. 15](#_Toc95570418)

[２２．Procedures for obtaining informed consent (including explanation items and methods) 15](#_Toc95570419)

[２３．Procedures to ensure that all requirements for research in situations of urgent and apparent life threatening situations are met. 15](#_Toc95570420)

[２４．Details of any financial burden or gratuity to the research subjects, if any 15](#_Toc95570421)

[In accordance with the administrative notice "Treatment of Publicly Funded Medical Care for Novel Coronavirus Infections" issued on March 4, 2020, this treatment is intended for patients who are COVID-19 positive, and since it will be conducted as publicly funded medical care, there will be no cost burden to the research subjects. 15](#_Toc95570422)

[２５．Response to adverse events and problems 16](#_Toc95570423)

[２６．Compensation for health damage and its details 17](#_Toc95570424)

[The drugs used in this study are those that are widely used in ordinary medical practice. In this sense, patients will not be exposed to risks that exceed those of routine medical care. Therefore, for any health damage caused by participation in this clinical trial, appropriate treatment according to the medical condition will be provided as insurance treatment, just as in normal medical care. In addition, the subjects of this study are COVID-19 positive cases, which are publicly funded medical care (issued on March 4, 2020, "Treatment of publicly funded medical care for novel coronavirus infection").In addition, no financial compensation such as a visitation fee or various allowances will be provided. 17](#_Toc95570425)

[２７．Nonconformity Report 17](#_Toc95570426)

[２８．Termination or discontinuation of the study 17](#_Toc95570427)

[２９．Handling of research results including incidental findings in cases where important findings may be obtained regarding the health or genetic characteristics of the research participants (policy on disclosure of research results, method of disclosure, etc.) 18](#_Toc95570428)

[３０．If part of the work related to the research is to be outsourced, the nature of the work and the method of supervision of the outsourcer 18](#_Toc95570429)

[３１．Possibility of using the samples and information obtained in this study for future research 18](#_Toc95570430)

[３２．Implementation system and procedures for monitoring and auditing 18](#_Toc95570431)

[３３．Attribution of intellectual property rights and ownership 18](#_Toc95570432)

[３４．Reference materials and bibliography 19](#_Toc95570433)

**A randomised controlled trial of teprenone in terms of preventing worsening of COVID-19 infections**

# １．Study title

　A randomised controlled trial of teprenone in terms of preventing worsening of COVID-19 infections

# ２．Organization of the research

　This research will be conducted under the following structure.

【Principal Investigator】

　　Eiki Ichihara

　 Department of Allergy and Respiratory Medicine, Okayama University Hospital

〒700-8558　2-5-1, Shikata-Cho, Kita-Ku, Okayama, Okayama, Japan

Tel：+81-86-235-7227

【Monitoring Manager】

　　Akihiko Taniguchi, Department of Allergy and Respiratory Medicine, Okayama University Hospital

# ３．Background, objective and significance of the study

**Background**

COVID-19 caused by SARS-CoV-2 has become a global pandemic with an exponential increase in the number of infections and deaths. 26% of patients hospitalized with COVID-19 are considered severe cases requiring intensive care unit admission1. Severe acute Severe acute respiratory infection (SARI) can be complicated by acute respiratory distress syndrome (ARDS), sepsis and septic shock, and multiple organ failure (Lancet Respir Med. 2020. Epub 2020/02/28). It is difficult to identify patients with COVID-19 who are expected to progress to SARI, and there is no established treatment to delay the progression to SARI. Although steroids have been administered to severe cases of SARI, findings from SARS and MERS suggest that steroids delay viral elimination and do not contribute to improved survival (PLoS Med 2006; 3: e343. Am J Respir Crit Care Med 2018; 197: 757 ); TNF-α and Th1/Th2 cytokines are elevated in severe cases of COVID-19 (Lancet 2020; 395: 497), suggesting that excessive stress, mainly cytokines, induces SAPK/JNK, resulting in cytotoxicity and leading to severe COVID-19 pneumonia Heat Shock Protein

　Heat shock proteins (HSPs) are a family of proteins that are up-regulated when cells are exposed to heat or other stress conditions and protect cells. Among them, HSP70 protects cells from damage caused by TNF-alpha (J Immunol 1993; 151: 4286) and reactive oxygen species (ROS) (FEBS Lett 1996: 391; 185).

It is known from preclinical data that teprenone (セルベックス®) has a cytoprotective effect by inducing HSP. In guinea pig gastric mucosal cells, teprenone has been shown to induce HSP60, 70, and 90 and to have cytoprotective effects2, and in rat myocardium it has been shown to protect against cell damage caused by ischemia3. In a mouse model of interstitial pneumonia, oral teprenone induced HSP-70 in the lung, reduced macrophage inflammatory protein-2 (MIP-2) and inflammatory cell infiltration, and inhibited lung fibrosis.4 In addition, gefitinib is known to cause drug-induced pneumonitis as an important adverse event.5 In addition, gefitinib is known to cause drug-induced pneumonitis,5 and administration of gefitinib to a bleomycin interstitial pneumonia mouse model decreased HSP-70 expression in lung tissue and exacerbated interstitial pneumonia.6 On the other hand, administration of teprenone restored HSP-70 expression, suppressed exacerbation of interstitial pneumonia, and prevented fibrosis.6 6 Furthermore, in a model of radiation pneumonitis induced by thoracic irradiation in C57BL/6 mice, teprenone administration inhibited lung fibrosis by suppressing HSP-70 induction and epithelial-mesenchymal transition.7 Thus, teprenone has been reported to have lung protective effects in various lung injury models. In vitro, 1uM of teprenone induces HSP70 in (gastric mucosa) cells and has cytoprotective effects. In humans, a single 150mg dose of teprenone has a blood concentration Cmax of 3-7uM. There is no data on the blood concentration of teprenone 50mg x 3 times (standard dose), but considering the above blood concentration and the concentration at which it has cellular effects, it is possible that the standard dose will be sufficient to produce an effect.

Viral pneumonia is caused by not only direct damage caused by viral proliferation in infected cells but also damage to non-infected cells caused by ROS from infiltrating neutrophils and macrophages. In addition to cytokines, control of cytotoxicity caused by ROS such as superoxide and NO is thought to be important in reducing the severity of the disease. Thioredoxin-1 (TRX-1) is a protective protein induced under stress conditions, and is an antioxidant enzyme with an SH group that has redox activity. it has cytoprotective effects against ROS, inhibits migration and adhesion of neutrophils and macrophages, suppresses AP-1 and NF-b, and inhibits complement activation. Yahsiro et al. demonstrated in an animal model that TRX-1 treatment reduced cytokines, neutrophil infiltration, and tissue damage in influenza pneumonia (Crit Care 2009; 61:303). In addition to HSP70, GGA has the ability to induce this TRX-1.

Based on the above basic findings, it is suggested that teprenone may reduce the severity of COVID-19 pneumonia. Other non-steroidal drugs currently in development include the anti-influenza drug favipiravir, the anti-HIV drug lopinavir-ritonavir, as well as Nafamostat mesylate and Camostat mesylate and Camostat, which inhibit viral receptors. All of these therapies target viruses and have different mechanisms of action from the cytoprotective action of teprenone, so they are expected to be effective in combination.

**Objective**

To determine whether teprenone is effective in reducing the severity of disease in patients with COVID-19.

**Significance**

If we can prevent COVD-19 pneumonia from becoming severe, we can not only reduce the number of potentially fatal cases, but also reduce the burden on the medical system by decreasing the number of ventilator and ECMO requirements, which will help prevent medical collapse.

# ４．Methods

（１）Type and design of the study

　　　Open-label, randomized, controlled, phase II study

（２）Outline of the study（Please see the flowchart of the study）

　　Eligibility should be confirmed in light of the selection and exclusion criteria, and written consent should be obtained from the patient. Considering that COVID-19 is highly infectious and that it is essential to avoid the spread of infection, the method of obtaining written consent should be conducted as described in "12. Procedures for obtaining informed consent

After pre-enrollment testing (screening tests), final confirmation of eligibility and enrollment. Outcomes will be assessed after the prescribed interventions.

＜Flowchart of the study＞


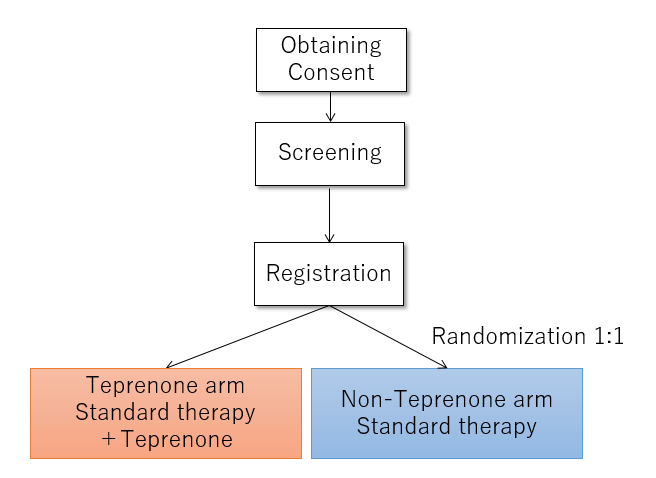


（３）Duration of intervention

　　 10days

（４）Overview of the intervention

　　Take 50 mg teprenone orally 3 times daily after meals. The dose should be administered continuously for 10 days until intolerable adverse events are observed. Generic products of the same drug may also be used.

Criteria for reduction of teprenone：The dose may be reduced or withdrawn at the discretion of the physician.　
る。しょうネル、加速度１チャンネル、上腕での非観血血圧の２２４ation 　If withdrawal is required for more than 7 days, this study treatment will be discontinued.

（５）How to manage the test drug

　　The drug to be used in this study is drug that have been approved for production and sale in Japan, and appropriate storage and other controls will be implemented based on the approved items for drugs.

（６）Provisions on concomitant therapy

１）Concomitant medication：No provision

２）Contraindicated drugs ：None other than teprenone in non-teprenone arm.

（７）Registration and allocation method

　　The principal investigator or sub-investigator will register and allocate patients according to the following procedures.

１）Patients who meet all the selection criteria and do not meet any of the exclusion criteria will be considered eligible cases, and written consent will be obtained. For details on how to obtain written consent for this study, please refer to "12. Procedures for obtaining informed consent".

1. Web registration using INDICE Cloud and allocation by minimization method (https://www.umin.ac.jp/indice/cloud.html). The settings for the minimization method are as follows: INDICE Cloud will issue a registration number and an allocation number.
2. Prepare a list of research subject identification codes (correspondence table) to be kept by the principal investigator, and describe the date of obtaining consent and the items necessary to correspond the research subject to the research subject identification code. The list shall include the registration number and allocation number issued by INDICE Cloud.
3. Record the research subject identification code and other information in the case list and case report form (CRF).
4. Treatment with or without teprenone based on the patient's research subject identification code.
5. If any withdrawal of consent, discontinuation, dropout, or deviation from the protocol occurs during the research period, it should be promptly reported to the principal investigator and the principal investigator (research secretariat). Upon receiving the report, the principal investigator will decide on an appropriate policy in consultation with the submitting physician and others, giving due consideration to the safety of the patient and the ethics of the research, and record the discussion in the medical record.
6. In case of incompleteness in registration or allocation, or inquiries regarding eligibility determination, the research secretariat will be contacted and the principal investigator will respond.

【Setting of the minimization method】

Age (over/under 70 years), gender, and comorbidities (hypertension, diabetes, cardiovascular disease, chronic lung disease, and cancer) will be used as allocation factors.

If the difference between the treatment group and the non-treatment group is more than 3, forced allocation to the lesser group will be performed, and if the difference is less than 3, random allocation will be performed.

（８）What to do after the research is completed

　　After the completion of this research, the principal investigator will provide the research subjects with medical care that he or she deems most appropriate, including the results obtained from this research.

# ５．Observation and test items

（１）Clinical characteristics of the enrolled patients

　　Age, gender, current medical history, comorbidities, previous medical history, smoking history, height, weight, and Concomitant medication/concomitant treatment

（２）Inspection Items

Blood count (WBC, white blood cell image)、

Biochemical test（AST、ALT、LDH、TP、Na、K、Cl、BUN、CRP、PCT、KL-6）

Chest X-ray、chest CT、SpO_2_

Biomarkers (using residual serum from routine blood collection) Comprehensive cytokine analysis using multiplex system, Hydropeoxides, TRX-1 and HMGB-1, HSP70, S100A8/A9

（３）Check for adverse events and side effects

The details of adverse events, time of onset and resolution, degree, treatment, outcome, severity assessment, and relationship to teprenone medication should be described in the CRF. If necessary, follow-up will be conducted until the time when isolation, including home isolation, is lifted.

| Item/period of inspection/investigation | Screening  (within 7 days before registration)Scre | Registration | Intervrnyion | | | | | follow up | | at the timing of intubation |
| --- | --- | --- | --- | --- | --- | --- | --- | --- | --- | --- |
|  |  |  | Day1 | Day3 | Day5 | Day7 | Day10 | Day14 | Day21 |  |
| Eligibility verification and registration |  | ● |  |  |  |  |  |  |  |  |
| COVID-19 PCR | ○ |  |  |  |  |  |  |  |  |  |
| clinical examination |  |  |  |  |  |  |  |  |  |  |
| blood count and biochemistry | ○ |  | ● |  | ▲ |  | ▲ | ▲ | ▲ | ▲ |
| KL-6・ | △ |  |  |  |  |  |  |  |  |  |
| procalcitonin |  |  |  |  |  |  |  |  |  |  |
| temprature | ○ | ● | ● | ● | ● | ● | ● | ▲ | ▲ | ● |
| dose of oxygen | 〇 |  | ● | ● | ● | ● | ● | ▲ | ▲ | ● |
| SpO2 | ○ |  | ● | ● | ● | ● | ● | ▲ | ▲ | ● |
| biomarker |  |  |  |  |  |  |  |  |  |  |
| Serum storage for biomarkersseru |  |  | ▲ |  | ▲ |  | ▲ |  |  |  |
| effect determination |  |  |  |  |  |  |  |  |  |  |
| chest X-ray | △ |  |  | ▲ |  |  | ▲ |  | ▲ | ▲ |
| chest CT | △ |  |  |  |  |  | ▲ |  | ▲ | ▲ |
| toxicity assessment |  |  |  |  |  |  |  |  |  |  |
| Subjective and subjective symptoms |  |  | ● | ● | ● | ● | ● | ● | ▲ | ▲ |

**【Schedule】**

**Patients who are discharged early will be observed within the observable period and do not need to come to**

**the hospital only for this study.**

△・▲ Not required, but recommended to be done whenever possible　　〇・△before registration、●・▲after registration

# ６．Evaluation Items

（１）Primary endpoint

　　Intubation rate

Indications for considering endotracheal intubation：

１．PaO2 less than 60 Torr under adequate oxygen therapy

２．Acute respiratory acidosis

３．Decreased level of consciousness, odd breathing, dyspnea

（２）Secondary endpoint

　　①mortality

　　②time to fever resolution

Fever：equal to or more than 37.5℃

Alleviation of fever：To be below 37.5℃ for more than 24 hours without the use of antipyretics

　　③dose of oxygen

　　④radiographic change with CT

⑤improvement of blood test

⑥severity

⑦time to discharge

⑧time to resolution of symptoms

　　⑨adverse events（CTCAE* version 5.0）*: Common Terminology Criteria for Adverse Events

# ７．Target number of the included patients and rationale for setting

　　【Target number of the included patients 】100

【Rationale for the target number】

We calculated the probability of verifying a significant difference in the primary endpoint between the groups (statistical power) in this number of cases. When the endotracheal intubation rate in the non-treated group was set at 22% and that in the treated group at 10%, and α = 0.1 (one-sided), the statistical power was 0.6406.

The rate of endotracheal intubation in the non-treated group is based on the National Institute of Infectious Diseases (Japan), and according to information published on the Web, of 287 cases of COVID-19 infection as of March 9, 2020, information on invasive ventilation was available for 133 cases. Of these, 29 (22%) were reported to have undergone endotracheal intubation.（<https://bit.ly/3eiiMhg>）。

# ８．Data aggregation and statistical analysis methods

All entries (e.g., CRF entries, database creation, entries in related reports, etc.) will be made by either the principal investigator, the principal investigator, or a research collaborator authorized by the principal investigator. The principal investigator will ensure the accuracy, completeness, and timeliness of the data and all other required reports. Data reported in CRFs that are derived from source documents (e.g., patient medical records) must be consistent with those source documents. In the event of any discrepancies, the rationale for the discrepancy should be provided in writing, signed and dated by the principal investigator, and kept in the research subject's file. All data must be derived from the original source.

Data on patients who drop out due to withdrawal of consent during the course of the study will not be used. For patients who drop out for other reasons, the available data up to the time of dropout will be used. Extreme outliers in continuous variables will not be excluded from the statistical analysis. If it is considered necessary to change the original statistical analysis plan (see below), the principal investigator and sub-investigators will consult with each other before changing the statistical analysis plan. No interim analysis will be conducted.

（１）Population for analysis

The population for the analysis of efficacy will be defined as all patients who have been assigned by the minimization method and have not withdrawn their consent.

The safety analysis population will be defined as all patients who have received at least one dose of the assigned treatment. In other words, patients in the teprenone-treated group will be those who have received at least one dose of teprenone, and patients in the non-treated group will be those who have not received any dose of teprenone during their participation in the study.

（２）Analysis method

The obtained data is entered into a dedicated database for tabulation and analysis.

【Main analysis and decision criteria】

For the primary endpoint of endotracheal intubation rate, if the null hypothesis "the rate of endotracheal intubation in the non-treated group is equal to the rate of endotracheal intubation in the treated group" is rejected, it was judged to be effective. If not, it is considered invalid from a clinical medical standpoint. The significance level for the entire study is set at 10% on one side. No adjustment for multiplicity will be made.

【Efficacy analysis 】

The analysis of efficacy is based on the treatment assigned to the patients by the minimization method according to the intention-to-treat (ITT) principle. An ITT analysis is defined as a method of analysis based on the assignment of a treatment group to a treatment or non-treatment group that is determined prior to the initiation of the intervention, rather than on the actual intervention performed. For example, patients who are assigned to the treatment group but never receive treatment for some reason are treated as the treatment group.

Analysis of the primary endpoint (endotracheal intubation rate)

1. Method of main analysis

Based on the observed endotracheal intubation rate, a binomial test will be performed for the null hypothesis that "the rate of endotracheal intubation in the non-treated group is equal to the rate of endotracheal intubation in the treated group. The competing hypothesis will be "the tracheal intubation rate of the treated group is less than that of the non-treated group". If the null hypothesis is rejected, the test is considered valid; if not, the test is considered clinically invalid. The risk ratio is calculated by dividing the risk of tracheal intubation in the treated group by the risk of tracheal intubation in the non-treated group. The exact 90% confidence interval based on the binomial distribution will be used to estimate the interval of the risk ratio.

1. Subgroup analysis will be conducted based on the factors described below.

＜Factors for which subgroup analysis is planned＞

Under 70 years old / Over 70 years old ・ Male / Female ・ Smoking history/No smoking history

Analysis of secondary endpoints (mortality)

The analysis will be performed in the same way as for the primary endpoint.

【Safety analysis】

Calculate the number and percentage of patients in the CTCAE version 5.0 Grade 1 to Grade 5 groups in the treated and non-treated groups, respectively.

# ９．Research period

　December 31, 2024 from the date of publication of the jRCT after approval by the accredited clinical research review committee. 　（Registration deadline: December 31, 2023）

# １０．Inclusion and exclusion criteria

（１）Inclusion criteria

　　All of the following criteria must be met.

1. Age 20 years or older on the date of obtaining consent
2. Diagnosis of COVID-19 is confirmed by PCR or antigen test.
3. Fever of 37.5 degrees or higher
4. Written consent to participate in this study.

（２）Exclusion criteria

　　Patient that fall under any of the following criteria is excluded from this study.

- - 1. Patients with a history of teprenone medication within 2 weeks prior to the planned start of treatment
    2. Patients who require ventilator management or extracorporeal membrane artificial lung (ECMO) management
    3. Patients with apparently active infectious diseases other than SARS-CoV-2 (Nontuberculous mycobacterial infection that does not require treatment or has not progressed for more than one month even with treatment can be enrolled.
    4. Pregnant or lactating patients
    5. Patients whose identity information is not available

# １１．Background and scientific rationale for the study (rationale and validity of the study)

Background

As described previously (3. Background, objective and significance of the study), we are in the midst of a global pandemic of COVID-19, and the development of treatment to prevent severe disease as well as the development of antiviral drugs is an urgent issue. Treatment for prevention of severe disease is a therapeutic strategy that should be used in combination with antiviral therapy to be developed in the future. Therefore, a treatment method with as few adverse events as possible and easy access to therapeutic agents would be ideal.

Rationale and validity of the study

The purpose of this study was to investigate the effect of teprenone on the prevention of aggravation of COVID-19. We believe that a randomized controlled trial with and without teprenone is a reasonable study design to achieve the purpose of this study.

Fever is one of the major symptoms of COVID-19, and the time to fever resolution is a reasonable primary endpoint.

A network of five major public hospitals, including Okayama University Hospital (Okayama Medical Cooperation Promotion Council), a clinical trial network in the Chugoku-Shikoku region, and designated medical institutions for Class II infectious diseases in the Kanto and Kinki regions are planned as the research system, and an appropriate system is in place to register the planned 100 cases.

Although it is difficult to accurately predict the number of COVID-19 cases, we believe that the planned number of cases can be enrolled within the study period, because more than 2,600 cases have been observed in Japan as of April 3, 2020, and the number of cases is rapidly increasing.

# １２．Procedures for obtaining informed consent

　The principal investigator or sub-investigator will provide the research subject with an explanation document that has been approved by an accredited clinical research review committee, and will provide sufficient written and oral explanations. Normally, free consent will be obtained in writing, but in this study, from the viewpoint of infection prevention, if free verbal consent is obtained from the research subject, a consent form will be prepared by the physician and a nurse or other witness who is not involved in the study. If the research subject recovers from COVID-19 infection, re-consent will be obtained whenever possible when all isolation restrictions, including home isolation, are no longer necessary.

When information on efficacy and safety, etc., that may affect the consent of research subjects is obtained, or when changes are made to the implementation plan, etc., that may affect the consent of research subjects, information will be promptly provided to the research subjects, and the will of the research subjects as to whether or not to participate in the research, etc., will be confirmed in advance. At the same time, the explanatory documents and consent documents, etc. will be revised with the approval of the Accredited Clinical Research Review Committee, and the research subject's consent will be obtained again. At that time, if the research subject has not yet recovered from COVID-19 infection, the method of obtaining consent shall be the same as the method of obtaining consent the first time.

The consent document includes the following information

①The name of the specific clinical research to be conducted, the fact that approval has been obtained from the administrator of the medical institution for the conduct of the specific clinical research, and the fact that an implementation plan has been submitted to the Minister of Health, Labour and Welfare.

②The name of the medical institution where the research is to be conducted and the name and title of the principal investigator (in the case where the specified clinical research is to be conducted as a multi-center collaborative research, including the name and title of the principal investigator, the name of the other medical institution, and the name and title of the principal investigator of said medical institution).

③Reason for being selected as a subject of the study

④Anticipated benefits and disadvantages of the study

⑤Refusal to participate in the study is possible

⑥Matters concerning withdrawal of consent

⑦A statement that no disadvantage will be caused by refusal to participate in the study or withdrawal of consent.

⑧How to disclose information about research

⑨The fact that the research subject or his/her representative may obtain or inspect the research protocol and other materials related to the conduct of the specified clinical research upon request, and the method of obtaining or inspecting such materials.

⑩Matters concerning the protection of personal information of research subjects

⑪Methods of storage and disposal of samples, etc.

⑫Involvement of the pharmaceutical product manufacturer or seller in the study and the details thereof

⑬Contact for responding to complaints and inquiries

⑭Matters related to expenses

⑮Availability of other treatments and comparison with anticipated benefits and disadvantages of other treatment

⑯Matters concerning compensation for health damage and provision of medical care

⑰Matters to be reviewed by the Accredited Clinical Research Review Committee, which provides review and opinion services for research and other matters related to the Authorized Clinical Research Review Committee for the study

⑱Other matters necessary for the implementation of the study

# １３．Handling of personal information, etc. (including methods of anonymization)

All researchers involved in this research will conduct it in compliance with the Declaration of Helsinki and the Clinical Research Act. When handling information related to the implementation of the research, a unique research ID will be assigned to each research subject, and a list of correspondences between names and research IDs will be created. When sending information to the research secretariat, etc., an identification number will be used, and sufficient care will be taken to ensure that the research subject's name, date of birth, and other information is not leaked outside the hospital. Names will be deleted from the original data and used for research. Throughout the research period, the corresponding table files will be password protected and stored securely to prevent leakage. The person responsible for the management of materials, samples, and personal information at each institution will follow the procedures of each institution.

When publishing the results of research, do not include information that can immediately identify the research subjects, such as names and dates of birth. In addition, do not use the information on research subjects obtained in the research for purposes other than those of the research.

# １４．Burdens, anticipated risks, and benefits to research subjects, a comprehensive assessment of these, and measures to minimize such burdens and risks

（１）Anticipated benefit

　　There is no direct benefit from participating in this study. Research results may contribute to future medical advances.

（２）Anticipated disadvantages (side effects)

　Study subjects will be required to take oral teprenone for 10 days. There is a possibility that the study treatment may cause illness. According to the package insert of the investigated drug, the most common adverse reactions are increased AST/ALT (less than 0.1-5%), and other adverse reactions such as constipation, diarrhea, nausea, dry mouth, abdominal pain, abdominal distention, headache, rash, pruritus, increased total cholesterol, and

Eyelid redness and burning sensation are all considered to have a probability of less than 0.1%.

（３）Response to research subjects in the event of an adverse event

When an adverse event is recognized, the principal investigator or sub-investigator should immediately take appropriate measures and record the event in the medical record and case report form. If the administration of the study drug is discontinued, or if treatment for the adverse event becomes necessary, the research subject will be informed.

（４）Changes to the research protocol, etc.

Information necessary for the safe conduct of clinical research will be collected and reviewed. If new safety information is obtained, the research protocol and consent explanatory document will be revised as necessary. Any changes or revisions to the research protocol or consent explanatory document will require prior approval from the Accreditation Clinical Research Review Committee. When changes are made to the research plan, the following notification form shall be submitted to the Minister of Health, Labor and Welfare.

①Notification of changes to the protocol (Ministerial Order Form No. 2)

②Protocol after change

When the following minor changes are made to the protocol, the changes shall be notified to the accredited clinical research review committee within 10 days of the change. In addition, a notification form (Ministerial Order Form No. 3) shall be submitted to the Minister of Health, Labor and Welfare.

①A change in the name of the person engaged in the research that does not involve a change in the person engaged in the study.

②Changes due to a change in the name of the region or a change in the lot number

（５）Discontinuation criteria for individual research subjects

【What to do when the study is discontinued】

When the principal investigator or sub-investigator judges that it is impossible to continue the research on an individual research subject for any of the reasons listed below, the research on that research subject will be discontinued. In such cases, the reason for discontinuation will be explained to the research subjects as necessary. Regarding the treatment of the research subjects after the discontinuation, we will respond in good faith so as not to disadvantage the research subjects.

【Discontinuance criteria】

①If the research subject declines to participate in the research or withdraws his/her consent

②If the continuation of this study is judged to be difficult due to the progression of COVID-19 or its complications.

③Intolerable adverse events

④In case the study itself is discontinued

⑤If, for any other reason, the principal investigator or sub-investigator decides that it is appropriate to discontinue the research（In this case, record the reason for the discontinuation.）

【What to do when study treatment is discontinued】

If the principal investigator or sub-investigator determines that it is not possible to continue the study treatment for an individual research subject for any of the reasons listed above, the investigator or sub-investigator will discontinue the study treatment for that research subject. In this case, the reason for discontinuation will be explained to the research subject as necessary. In addition, the treatment of the research subject after discontinuation will be handled in good faith so as not to disadvantage the research subject.

Of the patients who discontinued study treatment, those who received the study drug will continue to be observed for safety evaluation.

# １５．Method of storage and destruction of information including storage period

The principal investigator shall prepare a record of the following items

①Items that identify the subjects of this research

②Matters related to medical treatment and examination of the subjects of this research

③Matters related to participation in this study

④Other matters necessary to carry out this research

The samples and information collected in this study will be stored in a lockable place (at a location designated by the implementing medical institution) until five years after the end of the study, after which they will be disposed of with due care for personal information. Information that can immediately identify individuals, such as names, addresses, and dates of birth, should be deleted from the stored information.

The principal investigator shall keep the essential documents related to the implementation of the research, etc. (research protocol, implementation plan, documents related to the explanation to the subjects of this research and their consent, summary report and other documents prepared by the principal investigator pursuant to the provisions of the ministerial ordinance, or copies thereof, documents related to the review opinion work received from the authorized clinical research review committee, documents related to monitoring, list of identification codes for research subjects, consent forms, copies of case reports, etc., and other documents or records necessary to ensure the reliability of the data, etc.) for five years after the completion of the research. (documents related to monitoring, lists of identification codes for research subjects, consent forms, copies of case report forms, and other documents or records necessary to ensure the reliability of data, etc.) shall be stored in a lockable place (at a location designated by each medical institution) until five years after the end of the research, after which they shall be disposed of with sufficient care to protect personal information. After that, dispose of them with care. Information on the computer should be completely deleted, and paper media (materials) should be shredded and disposed of.

# １６．Matters related to access to source documents, etc.

In this study, source documents are defined as the following.

①Records of researcher consent and information provision

②Medical records, clinical examination data and imaging films, etc., data at the time of case registration, and the original records of case reports

The principal investigator and the implementing medical institution will provide direct access to all clinical research-related records, including source documents, during monitoring and auditing related to clinical research, as well as during investigations by accredited clinical research review committees and regulatory authorities.

# １７．Periodic reporting

The Principal Investigator shall, in principle, report to the Accredited Clinical Research Review Committee on the following details concerning the implementation of this research every year from the date of submission of the protocol to the Minister of Health, Labor and Welfare, and within two months after the expiration of said period. Use the Uniform Form 5 Periodic Report and Appendix Form 3 Periodic Report.

①Number of research subjects who participated in this study

②Occurrence and subsequent progress of severe adverse effects related to this study

③Occurrence of noncompliance with the ministerial ordinance or research protocol pertaining to this research and subsequent actions

④Evaluation of the safety and scientific validity of this study

⑤Matters concerning the involvement of pharmaceutical manufacturers and sellers, etc. in this research

In addition, within one month from the date on which the Accredited Clinical Research Review Committee gives its opinion, the following information shall be reported to the Minister of Health, Labor and Welfare using the attached Form 3.

①Name of the authorized clinical research review committee described in the implementation plan

②Approval or disapproval of the continuation of this research by the Accredited Clinical Research Review Committee

③Number of subjects who participated in this study

　The principal investigator shall provide information to the principal investigator regarding the contents of the periodic report and its approval by the committee and submission to the Minister of Health, Labor and Welfare. In addition, the principal investigator and each principal investigator shall report to the administrators of their respective medical institutions by means of the aforementioned periodic reports.

# １８．Status of conflicts of interest related to research and other research, including sources of funding for research, conflicts of interest related to research for the duration of the research, and personal earnings

This research is planned to be carried out with 5 million yen from the Okayama University Academic Fund.

In addition, the principal investigator shall declare any conflicts of interest in accordance with the "Guidance for the Management of Conflicts of Interest in Clinical Research Law" and obtain its review and approval. In addition, whether there is any change in the conflict of interest of the research and the conflict of interest of individuals shall be checked at the time of periodic reporting and reported to the Accredited Clinical Research Review Committee.

# １９．Methods of disclosing information about research (registration of research plans and publication of research results)

This study is registered in the Japan Registry of Clinical Trials (jRCT: https://jrct.niph.go.jp/), a system for disclosing clinical research implementation plans and research summaries. The results obtained in this study will be presented on the jRCT and at various related conferences, such as the Japanese Society of Internal Medicine, and will be published as papers in professional journals.

# ２０．Response to consultation, etc. from research subjects, etc.

The Principal Investigator and Subcontracting Physicians will respond appropriately and promptly to consultations, inquiries, complaints, etc., from research subjects, their guardians, etc., and related persons.

Consultation Desk Manager

Eiki Ichihara

Department of Allergy and Respiratory Medicine

Okayama University Hospital

　　 700-8558　2-5-1, Shikata-Cho, Kita-Ku, Okayama, Okayama, Japan岡

　　Tel： 086-235-7227（Weekdays: 9:00 a.m. - 5:00 p.m.）

　　　　　 086-235-6765（Weekday nights and holidays）

　　FAX： 086-232-8227

# ２１．Procedures for obtaining informed consent from a surrogate, etc.

In this study, no surrogate will be established.

# ２２．Procedures for obtaining informed consent (including explanation items and methods)

　Not applicable for this study.

# ２３．Procedures to ensure that all requirements for research in situations of urgent and apparent life threatening situations are met.

　Not applicable for this study.

# ２４．Details of any financial burden or gratuity to the research subjects, if any

# In accordance with the administrative notice "Treatment of Publicly Funded Medical Care for Novel Coronavirus Infections" issued on March 4, 2020, this treatment is intended for patients who are COVID-19 positive, and since it will be conducted as publicly funded medical care, there will be no cost burden to the research subjects.

In addition, there will be no honorarium for participating in this study.

# ２５．Response to adverse events and problems

（１）Definition of adverse events and problems

Adverse effects refer to illness, disability, death, or infection suspected to be caused by the conduct of the specified clinical research, as well as malfunctions that could cause these.

（２）Severe adverse events

　Serious adverse events are defined as follows.

　１）Death or threatened death

　２）the following adverse events

①　Cases that require hospitalization or extension of hospital stay at a medical institution for treatment

②　Cases that cause permanent disability

③　Adverse events that may lead to disability, etc.

④　① to ③, and death and side effects that are as serious as those that may lead to death.

⑤　Congenital diseases or abnormalities in the later generations

（３）Causal relationship with research

The relationship of all adverse events to the conduct of the study or to the treatment under study will be determined by the principal investigator, principal investigator, or sub-investigator. In addition to the temporal relationship to the initiation of the study treatment, the determination will take into account the course of the underlying disease, complications, concomitant medications, study procedures, accidents, and other external factors.

（４）Predictability

Predictability of adverse events should be determined based on the package insert and interview form. If the nature, severity or frequency of an adverse event is inconsistent, it should be considered an unknown adverse event. Predictable adverse events and failures include elevated AST/ALT (<0.1-5%) and others (constipation, diarrhea, nausea, dry mouth, abdominal pain, abdominal distention, headache, rash, pruritus, elevated total cholesterol, eyelid redness/heat, etc.) all <0.1%.

（５）Outcome

The principal investigator and sub-investigators (principal investigators, etc.) will provide appropriate treatment and therapy for all illnesses that occur, and will conduct follow-up studies until symptoms disappear or laboratory values return to baseline or pre-administration values, or until the principal investigator, etc. determines that follow-up is not medically necessary. The outcome of adverse events and failures will be classified as follows: 1) recovery (disappearance or recovery of symptoms, findings, and laboratory values), 2) minor recovery (reduction of the event in question), 3) unrecovered (no change to worsening), 4) death, and 5) unknown.

（６）Responsibilities of principal investigators, etc. (reporting of serious adverse events)

① If a principal investigator, etc. becomes aware of the occurrence of a serious illness, etc. in the conduct of specified clinical research, he/she shall take the necessary measures, including explanation and treatment of the research subjects, etc.

② When a principal investigator, etc. becomes aware of the occurrence of a disease, etc., he/she shall report to that effect to the administrator of the medical institution and then, pursuant to the provisions of Ordinance of the Ministry of Health, Labour and Welfare, report to that effect to the accredited Clinical Research Review Committee described in the implementation plan of said specified clinical research using the Uniform Form 8.

③ When a principal investigator becomes aware of any matter concerning the occurrence of a disease, etc. suspected to be caused by the conduct of specified clinical research, which is specified by an Ordinance of the Ministry of Health, Labour and Welfare (see the table below), the Principal Investigator shall report to that effect to the Minister of Health, Labour and Welfare using the attached Form 2-1 (Drugs), as specified by an Ordinance of the Ministry of Health, Labour and Welfare.

| Predictability | Adverse events | Report to the Accredited Clinical Research Review Committee | Report to the Minister of Health, Labor and Welfare |
| --- | --- | --- | --- |
| Unpredictable | Death or may lead to death | within 7 days | within 7 days |
|  | other adverse events | within 15 days | within 15 days |
| Predictable | Death or may lead to death | within 15 days | at periodic reporting |
|  | other adverse events | at periodic reporting | at periodic reporting |

# ２６．Compensation for health damage and its details

# The drugs used in this study are those that are widely used in ordinary medical practice. In this sense, patients will not be exposed to risks that exceed those of routine medical care. Therefore, for any health damage caused by participation in this clinical trial, appropriate treatment according to the medical condition will be provided as insurance treatment, just as in normal medical care. In addition, the subjects of this study are COVID-19 positive cases, which are publicly funded medical care (issued on March 4, 2020, "Treatment of publicly funded medical care for novel coronavirus infection").In addition, no financial compensation such as a visitation fee or various allowances will be provided.

# ２７．Nonconformity Report

When the principal investigator becomes aware that the clinical research is not in compliance with laws and regulations or the research protocol (referred to as "noncompliance"), he/she shall promptly report it to the administrator of the research institution. When a research subcontracting physician becomes aware of nonconformity, he/she shall promptly report it to the principal investigator. When a investigator becomes aware of the occurrence of a serious adverse event, he must promptly notify the principal investigator. In addition, the principal investigator shall promptly provide the information to other investigators to that effect. In this case, said other investigators shall promptly report the contents of said information to the administrator of the medical institution. In the case that a nonconformity is found to be particularly serious, the principal investigator shall promptly obtain the opinion of an accredited clinical research review committee.

The term "serious nonconformity" refers to noncormity that may affect the human rights and safety of subjects of clinical research, as well as the progress of the research and the reliability of the results. For example, non-compliance with selection/exclusion criteria, discontinuation criteria, concomitant use of prohibited therapies, etc., does not include non-compliance with the research protocol to avoid immediate danger to the subjects of the clinical research or for other unavoidable medical reasons. If a serious noncompliance occurs, the principal investigator shall take measures to prevent its recurrence, inform the submitting investigator and other persons engaged in the clinical research concerned, and thoroughly prevent its recurrence. (3) At the time of regular reporting to the Committee, the occurrence of nonconformity with laws and regulations or the research protocol pertaining to the clinical research concerned and the subsequent response shall be reported.

# ２８．Termination or discontinuation of the study

（１）Termination of the study

　When the period for collecting data related to the primary endpoint is completed, a report on the primary endpoint shall be prepared within one year in principle, and when the period for collecting data related to all evaluation items described as matters related to the content of the clinical research is completed, a summary report and outline

A report on the primary endpoint or a summary report and its summary after hearing opinions from the Review Committee shall be submitted to the administrator of the medical institution.

In addition, the summary of the primary endpoint report or the summary report shall be published in the jRCT within one month from the date of the committee's opinion. The publication in the jRCT shall be reported to the administrator.

When submitting the summary report to the Minister of Health, Labour and Welfare, the research protocol and explanatory documents should also be submitted.

（２）Discontinuation of the study

The principal investigator will consider whether or not to continue the research if any of the following applies.

１）When significant information regarding the quality, safety, or efficacy of teprenone used in this study becomes available

２）When it is judged that it is difficult to achieve the planned number of cases due to difficulty in recruiting research subjects

３）When the purpose of the study is achieved before the expected number of patients or the expected duration is reached

４）When the Accredited Clinical Research Review Committee instructs changes to the implementation plan, etc., and it is judged to be difficult to accept the changes

If the Authorized Clinical Research Review Committee recommends or directs that the research be discontinued, the research shall be discontinued.

When the decision to discontinue the research is made, the decision shall be notified to the Accredited Clinical Research Review Committee described in the implementation plan of said specified clinical research within 10 days from the date of discontinuation using the Uniform Form 11, and the decision shall be notified to the Minister of Health, Labor and Welfare using Form 4. Even when the notification of discontinuation is submitted, the principal investigator shall submit reports on diseases, etc. and periodic reports, etc. until the clinical research is completed. In addition, until the completion of the specified clinical research, if the change corresponds to a change in the matters concerning the progress of the specified clinical research, notification of the change in the implementation plan shall be made. In addition, information on the discontinuation of the research shall be provided to the principal investigator. Each principal investigator shall respond in accordance with the regulations of each institution.

# ２９．Handling of research results including incidental findings in cases where important findings may be obtained regarding the health or genetic characteristics of the research participants (policy on disclosure of research results, method of disclosure, etc.)

Not applicable.

# ３０．If part of the work related to the research is to be outsourced, the nature of the work and the method of supervision of the outsourcer

No work related to this research will be outsourced to other organizations.

# ３１．Possibility of using the samples and information obtained in this study for future research

The information obtained in this study may be used in future studies, in which case the approval of the Ethics Committee will be obtained.

# ３２．Implementation system and procedures for monitoring and auditing

（１）Monitoring

　Monitoring will be conducted in this study. The specific procedures will be specified in a separate monitoring plan.

（２）Auditing

　Not conducted in this study.

# ３３．Attribution of intellectual property rights and ownership

There is a possibility that intellectual property rights and other rights may arise from this research. The handling of such rights will be discussed at Okayama University.

# ３４．Reference materials and bibliography

1. Wang D, Hu B, Hu C, et al. Clinical Characteristics of 138 Hospitalized Patients with 2019 Novel Coronavirus-Infected Pneumonia in Wuhan, China. *JAMA - J Am Med Assoc*. 2020. doi:10.1001/jama.2020.1585

2. Hirakawa T, Rokutan K, Nikawa T, Kishi K. Geranylgeranylacetone induces heat shock proteins in cultured guinea pig gastric mucosal cells and rat gastric mucosa. *Gastroenterology*. 1996. doi:10.1053/gast.1996.v111.pm8690199

3. Ooie T, Takahashi N, Saikawa T, et al. Single oral dose of geranylgeranylacetone induces heat-shock protein 72 and renders protection against ischemia/reperfusion injury in rat heart. *Circulation*. 2001. doi:10.1161/hc3901.095771

4. Fujibayashi T, Hashimoto N, Jijiwa M, Hasegawa Y, Kojima T, Ishiguro N. Protective effect of geranylgeranylacetone, an inducer of heat shock protein 70, against drug-induced lung injury/fibrosis in an animal model. *BMC Pulm Med*. 2009. doi:10.1186/1471-2466-9-45

5. Kudoh S, Kato H, Nishiwaki Y, et al. Interstitial lung disease in Japanese patients with lung cancer: A cohort and nested case-control study. *Am J Respir Crit Care Med*. 2008. doi:10.1164/rccm.200710-1501OC

6. Namba T, Tanaka KI, Hoshino T, Azuma A, Mizushima T. Suppression of expression of heat shock protein 70 by gefitinib and its contribution to pulmonary fibrosis. *PLoS One*. 2011. doi:10.1371/journal.pone.0027296

7. Kim JS, Son Y, Jung MG, et al. Geranylgeranylacetone alleviates radiation-induced lung injury by inhibiting epithelial-to-mesenchymal transition signaling. *Mol Med Rep*. 2016. doi:10.3892/mmr.2016.5121
