# Supplementary material for: A randomized controlled trial of teprenone in terms of preventing worsening of COVID-19 infection
Source: PLoS One. 2023 Oct 26;18(10):e0287501. doi: 10.1371/journal.pone.0287501 (PMC10602324; doi:10.1371/journal.pone.0287501)
Supplement: S2 Protocol — (DOCX) [file pone.0287501.s003.docx]

COVID-19肺炎の重症化抑制を目的としたテプレノン療法の

第II相ランダム化比較探索的臨床試験

研究実施計画書

【研究責任医師】

市原　英基

　岡山大学病院呼吸器・アレルギー内科

　住所：〒700-8558　岡山市北区鹿田町2-5-1

　電話番号：086-235-7227

　FAX番号：086-232-8226

【研究事務局】

　岡山大学病院呼吸器・アレルギー内科

　事務局担当　市原英基

　住所：〒700-8558　岡山市北区鹿田町2-5-1

　電話番号：086-235-7227（平日：9時～17時）

　　　　　　086-235-6765（平日夜間、休日）

　FAX番号：086-232-8226

【研究実施予定期間】

　認定臨床研究審査委員会承認後のjRCT公表日から2024年12月31日

2020年4月8日　第1.0版作成

2020年8月17日　第2.0版作成

2020年10月26日　第2.1版作成

2020年12月26日　第2.2版作成

2021年5月21日　第3.0版作成

目次

[１．研究の名称 3](#_Toc37888296)

[２．研究の実施体制（研究機関の名称および研究者等の氏名を含む） 3](#_Toc37888297)

[３．研究の背景、目的、意義 3](#_Toc37888298)

[４．研究の方法 4](#_Toc37888299)

[５．観察および検査項目 7](#_Toc37888300)

[６．評価項目 9](#_Toc37888301)

[７．目標症例数および設定根拠 9](#_Toc37888302)

[８．データの集計および統計解析方法 9](#_Toc37888303)

[９．研究実施期間 11](#_Toc37888304)

[１０．研究対象者の設定方針 11](#_Toc37888305)

[１１．研究の背景および科学的合理性の根拠（研究の合理性・妥当性） 11](#_Toc37888306)

[１２．インフォームド・コンセントを受ける手続き 12](#_Toc37888307)

[１３．個人情報等の取扱い（匿名化の方法を含む） 12](#_Toc37888308)

[１４．研究対象者に生じる負担、予測されるリスク及び利益、これらの総合的評価並びに当該負担及びリスクを最小化する対策 13](#_Toc37888309)

[１５．情報の保管及び破棄の方法（保管期間を含む） 14](#_Toc37888310)

[１６．原資料等の閲覧に関する事項 14](#_Toc37888311)

[１７．定期報告 14](#_Toc37888312)

[１８．研究の資金源、研究期間の研究に係る利益相反及び個人の収益等、研究等の研究に係る利益相反に関する状況 15](#_Toc37888313)

[１９．研究に関する情報公開の方法（研究計画の登録および研究結果の公表） 15](#_Toc37888314)

[２０．研究対象者等からの相談等への対応 15](#_Toc37888315)

[２１．代諾者等からのインフォームド・コンセントを受ける場合の手順 15](#_Toc37888316)

[２２．インフォームド・アセントを得る手続き（説明事項、説明方法含む） 15](#_Toc37888317)

[２３．緊急かつ明白な生命の危機が生じている状況での研究に関する要件の全てを満たしていることを確認するための手順 15](#_Toc37888318)

[２４．研究対象者等に経済的負担又は謝礼があればその内容 16](#_Toc37888319)

[２５．疾病等および不具合が発生した場合の対応 16](#_Toc37888320)

[２６．健康被害に対する補償の有無及びその内容 17](#_Toc37888321)

[２７．不適合報告 17](#_Toc37888322)

[２８．研究の終了、中止 17](#_Toc37888323)

[２９．研究対象者の健康、遺伝的特徴に関する重要な知見が得られる可能性がある場合の研究結果（偶発的所見を含む）の取扱い(研究結果の開示の方針、開示の方法等) 18](#_Toc37888324)

[３０．研究に関する業務の一部を委託する場合には、当該業務内容及び委託先の監督方法 18](#_Toc37888325)

[３１．本研究で得られた試料・情報を将来の研究に用いる可能性 18](#_Toc37888326)

[３２．モニタリング及び監査の実施体制及び実施手順 18](#_Toc37888327)

[３３．知的財産権、所有権の帰属先 18](#_Toc37888328)

[３４．参考資料・文献リスト 18](#_Toc37888329)

COVID-19肺炎の重症化抑制を目的としたテプレノン療法の第II相ランダム化比較探索的較試験

研究実施計画書

# １．研究の名称

　COVID-19肺炎の重症化抑制を目的としたテプレノン療法の第II相ランダム化比較探索的較試験

# ２．研究の実施体制（研究機関の名称および研究者等の氏名を含む）

　本研究は以下の体制で実施する。

【研究代表医師】

　　所属：岡山大学病院呼吸器・アレルギー内科　職名：講師　氏名：市原英基

〒700-8558　岡山県岡山市北区鹿田町2-5-1

電話：086-235-7227

【研究責任医師】（別紙リストを参照）

　【連絡先】

　　岡山大学病院呼吸器・アレルギー内科　研究事務局担当　市原英基

　　住所：〒700-8558　岡山市北区鹿田町2-5-1

　　電話番号：086-235-7227（平日：9時～17時）

　　　　　　　086-235-6765（平日夜間、休日）

　　FAX番号：086-232-8226

【モニタリング責任者】

　　所属：岡山大学病院呼吸器・アレルギー内科　職名：助教　氏名：谷口暁彦

# ３．研究の背景、目的、意義

**研究の背景**

SARS-CoV-2によるCOVID-19発症は感染者数・死亡者数ともに指数関数的に増加し現在世界的なpandemicとなっている。**COVID-19入院患者のうち、26%は集中治療室入室を要する重症例**とされる^1^。 重症急性呼吸器感染症（Severe acute respiratory infection; SARI）へ進展する症例では、急性呼吸器疾患症候群（ARDS）、敗血症および敗血症性ショック、多臓器不全を合併することがある（Lancet Respir Med. 2020. Epub 2020/02/28）。 COVID-19患者のうちSARIへの進展が予期される症例の把握は困難であり、またSARIへの進展を遅らせる治療は確立していない。 SARIの重篤例にはステロイドが投与されているものの、SARSやMERSからの知見ではステロイドはウイルス排除を遅らせ、生存率の向上に寄与しないとされている（PLoS Med 2006; 3: e343. Am J Respir Crit Care Med 2018; 197: 757）。COVID-19の重篤例ではTNF-αやTh1/Th2サイトカインが上昇しており（Lancet 2020; 395: 497）、サイトカインを中心とした過剰なストレスがSAPK/JNKを誘導した結果、細胞障害が進みCOVID-19肺炎の重篤化に繋がると考えられる。

　熱ショックタンパク質（Heat Shock Protein; HSP）は細胞が熱等のストレス条件下にさらされた際に発現が上昇して細胞を保護するタンパク質の一群であり、分子量によりHSP60、HSP70、HSP90などのファミリーがある. なかでもHSP70はTNF- α （J Immunol 1993; 151: 4286)や活性酸素（Reactive oxygen species; ROS）（FEBS Lett 1996: 391; 185）による障害から細胞を保護する。

テプレノン**（セルベックス®）**は、HSP誘導による細胞保護作用があることが前臨床のデータから知られている。モルモット胃粘膜細胞においてHSP60、70、90を誘導し細胞保護作用を示すことが確認されており^2^、ラットの心筋では虚血による細胞ダメージからの保護効果を示している^3^. 肺疾患に対するテプレノンの保護作用も数多く報告されており、ブレオマイシンによる間質性肺炎マウスモデルでは、テプレノン経口投与により肺内のHSP-70を誘導し、macrophage inflammatory protein-2 (MIP-2)・炎症細胞浸潤を減少させ、肺の線維化を抑制したと報告されている^4^。また、ゲフィチニブは、重要な有害事象として薬剤性肺臓炎が知られ^5^、ブレオマイシン間質性肺炎マウスモデルにゲフィチニブを投与すると肺組織におけるHSP-70の発現が減少し間質性肺炎を悪化させる^6^。一方、テプレノン投与した場合、HSP-70の発現が回復し間質性肺炎の悪化を抑え線維化を予防したと報告されている^6^。さらに、C57BL/6マウスへの胸部照射による放射線肺炎モデルでは、テプレノン投与によりHSP-70誘導および上皮間葉系移行を抑制することで肺の線維化を抑制したと報告されている^7^。以上より、様々な肺障害モデルにおいて、テプレノンによる肺保護作用が報告されている。in vitroでは　テプレノン1uMで（胃粘膜）細胞にHSP70を誘導・細胞保護作用があることが示されている。またヒトでは、テプレノン　150mg単回投与で血中濃度Cmaxは3-7uMとなっている。今回用いるテプレノン50mgx3回投与（通常用量）での血中濃度データはないが、上記血中濃度と細胞効果を及ぼす濃度を鑑みると通常用量で充分効果を出す可能性がある。

ウイルス性肺炎は、感染細胞中のウイルス増殖によってもたらされる直接障害以外に、浸潤した好中球・マクロファージからのROSによる非感染細胞への障害が加わることにより重篤化する。 サイトカインと並んでスーパーオキシドやNOなどのROSによる細胞障害を制御することは重篤化を抑制する上で重要と考えられる。チオレドキシン（Thioredoxin-1：TRX-1）は、ストレス条件下で誘導される防御蛋白で、酸化還元活性を有するSH基を持つ抗酸化酵素である。ROSからの細胞保護作用を示すほか、好中球・マクロファージの遊走・接着の抑制、AP-1やNF-κb の抑制、補体活性化抑制などの抗炎症作用を有する（Adv Drug Deliv Rev 2009; 61:303）。Yahsiroらは動物モデルを使い、TRX-1投与によりインフルエンザ肺炎のサイトカイン、好中球浸潤、組織障害を軽減できることを明らかにした（Crit Care Med 2013; 41:171）。GGAはHSP70に加え、このTRX-1の誘導作用を有している。

以上の基礎的な知見をもとにテプレノンがCOVID-19肺炎の重篤化を抑制する可能性が示唆される。現在、開発されているステロイド以外の治療薬として、抗インフルエンザウイルス薬ファビピラビル（アビガン®）、抗HIV薬ロピナビル・リトナビル（カレトラ®）、さらにウイルス感染のレセプターを阻害するNafamostat mesylate（フサン）、Camostat mesylate（フォイパン）があげられる。いずれもウイルスをターゲットとした治療法であり、テプレノンの細胞保護作用と作用機序が異なるため、併用効果が期待される。

**目　的**

COVID-19患者に対し、テプレノン投与による重症化抑制効果があるかどうかを検討する．

**意　義**

COVD-19肺炎の重症化を予防することができれば、致死的となり得る潜在的重症症例を抑制できるだけでなく、人工呼吸器必要数・ECMO必要数の減少による医療体制への負荷軽減ができ、医療崩壊の予防につながる。

# ４．研究の方法

（１）研究の種類・デザイン

　　　非盲検、ランダム化比較、第II相試験

（２）研究のアウトライン（研究のフローチャート参照）

　　選択基準・除外基準に照らし合わせて適格性の確認を行い、患者から文書同意を取得する。なお、COVID-19は高感染力であること、感染拡大を回避することが必須であることを考慮し、文書同意の取得方法については「12. インフォームド・コンセントを受ける手続き」に記載したとおりに実施する。

登録前検査（スクリーニング検査）の後、適格性を最終的に確認し、登録を行う。規定の介入の後、アウトカムの評価を行う。


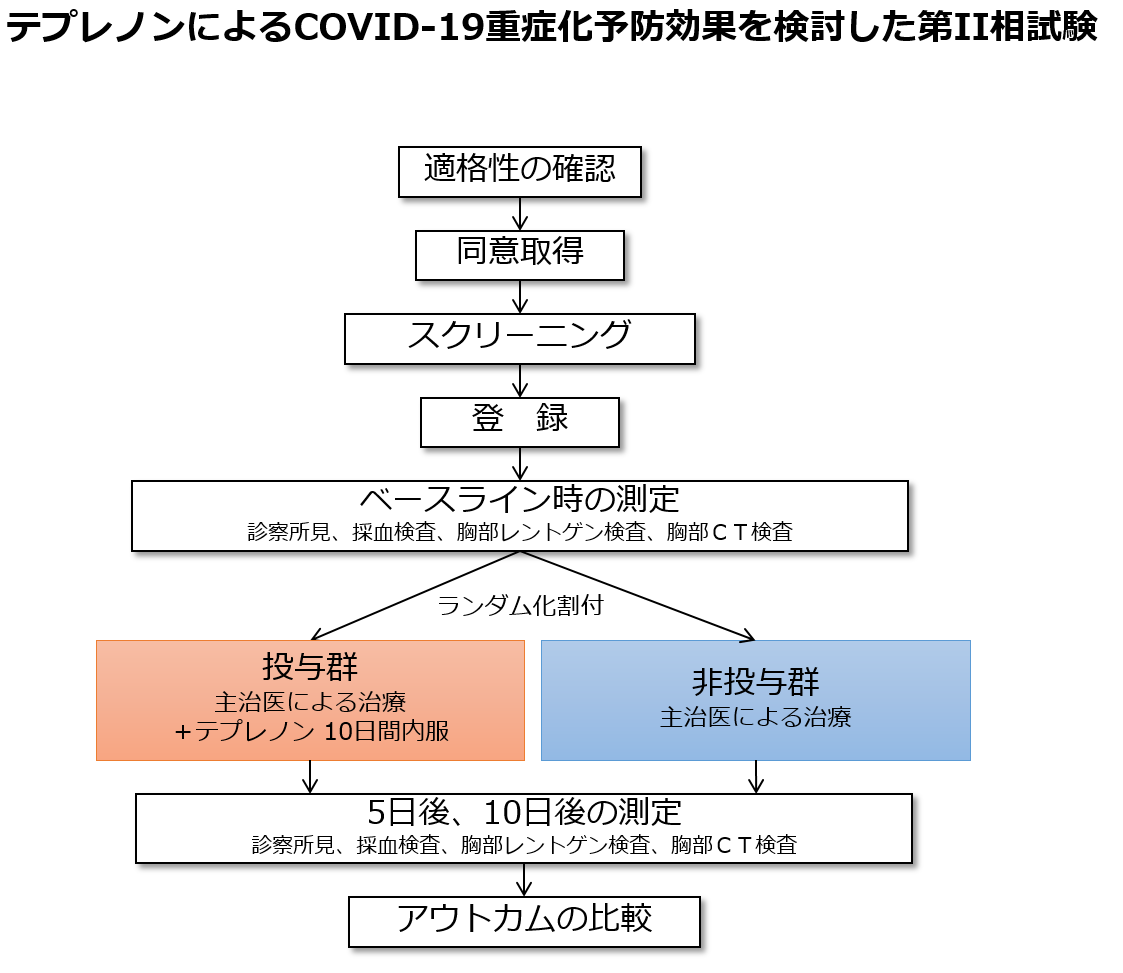
＜研究のフローチャート＞

（３）研究対象者の研究参加予定期間（患者での介入期間）

　　２８日間（スクリーニング期：０～７日間、介入期間：１０日間、後観察期間：１１日間）

（４）介入する医薬品・医療機器の概要とその介入内容

　　①概要

| 一般名 | テプレノン |
| --- | --- |
| 商品名／製造販売業者 | セルベックスカプセル50mg^🄬^・セルベックス細粒10%^🄬^　（エーザイ株式会社）、 |
| 剤形・性状・含量 | 上半分灰青緑色不透明・下半分淡橙色不透明のカプセル製剤あるいは白色〜帯黄白色の細粒剤で、カプセル製剤1カプセル中にテプレノン50mgあるいは細粒剤0.5gにテプレノン50mg含有する. |
| 薬効分類 | 胃炎・胃潰瘍治療剤 |
| 効能・効果 | 下記疾患の胃粘膜病変（びらん、出血、発赤、浮腫）の改善  急性胃炎、慢性胃炎の急性増悪期 |
| 用法・用量 | カプセル50mg  通常成人、3カプセル（テプレノンとして150mg)を1日3回に分けて食後に経口投与する。  なお、年齢、症状により適宜増減する.  細粒10％  通常成人、細粒1.5g（テプレノンとして150mg)を1日3回に分けて食後に経口投与する.  なお、年齢、症状により適宜増減する. |
| 保管条件 | 室温保存 |

＊同薬剤のジェネリック製品でも使用可能とする

　　②介入内容

セルベックスカプセル50mg^🄬^１カプセルまたは セルベックス細粒10%^🄬^　0.5gを、1日3回食後、経口投与する。忍容できない有害事象を認めるまで10日間、連続投与する. なお、同薬剤のジェネリック製品でも使用可能とする。

＜使用可能なジェネリック製品＞

セルテプノンカプセル50mg（武田テバ薬品）、セルテプノン細粒10%（武田テバ薬品）

テプレノンカプセル50mg「トーワ」（東和薬品）

テプレノン細粒10％「トーワ」（東和薬品）

テプレノンカプセル50mg「日医工」（日医工）、テプレノン細粒10％「日医工」（日医工）

テプレノンカプセル50mg「サワイ」（サワイ）、テプレノン細粒10％「サワイ」（サワイ）

テプレノンカプセル50mg「アメル」（共和薬品工業）

テプレノン細粒10％「アメル」（共和薬品工業)

テプレノンカプセル50mg「テバ」（武田テバファーマ）

テプレノンカプセル50mg「YD」（陽進堂）、テプレノン細粒10％「YD」（陽進堂）

テプレノンカプセル50mg「武田テバ」（武田テバ薬品）

デムナロンカプセル50mg（鶴原製薬）、デムナロン細粒10％（鶴原製薬）

・休薬/減量基準：担当医師の判断により適宜休薬/減量可能とする。

る。しょうネル、加速度１チャンネル、上腕での非観血血圧の２２４ation 　 ただし、7日間を超えて休薬が必要な場合、本試験治療は中止とする。

（５）被験薬の管理方法

　　本研究で用いる薬剤は国内において製造販売承認を取得している医薬品であり、医薬品等の承事項に基づく適切な保管等の管理を行う。

（６）併用薬（療法）等に関する規定

１）併用薬（療法）：規定しない

２）併用禁止薬（療法）：規定しない

（７）症例登録、割付方法

　　研究責任医師または研究分担医師は、以下の手順で症例登録および割付を行う。

１）すべての選択基準を満たし、除外基準のいずれにも合致しない患者を適格例とし、文書による同意を取得する。なお、本試験の文書同意の取得方法については、「12. インフォームド・コンセントを受ける手続き」を参照すること。

1. INDICE Cloudを用いたweb登録および最小化法で割付けを行う（https://www.umin.ac.jp/indice/cloud.html）。最小化法の設定は下記の通りである。INDICE Cloudからは、登録番号、割付番号が発行される。
2. 研究責任医師が保管する研究対象者識別コードリスト（対応表）を作成し、同意取得日および研究対象者と研究対象者識別コードを対応させるために必要な事項を記載する。記載事項には、INDICE Cloudから発行された登録番号、割付番号を含む。
3. 研究対象者識別コード等を症例一覧表および症例報告書（case report form：CRF）に記録する。
4. 患者の研究対象者識別コードに基づいて、テプレノンの投与あり治療または非投与での治療を行なう。
5. 研究期間中に、同意撤回、中止、脱落、プロトコールからの逸脱等が生じた時は、速やかに研究責任医師及び研究代表医師（研究事務局）に報告する。報告を受けた研究責任医師は患者の安全性、研究の倫理性を十分に配慮した上で、研究分担医師らと協議の上、適切な方針を決定し、診療録等に協議内容を記録する。（別紙）
6. 登録・割付に不備が生じた場合および適格性判断に関する問い合せは、研究事務局に連絡し、研究代表医師が対応する。（別紙）

【最小化法の設定】

割付因子として、年齢（70歳以上/70歳未満）・性別・合併症（高血圧・糖尿病・心血管疾患・慢性肺疾患・がん）を用いる。

投与群と非投与群との差が3以上の場合は少ない群に強制割付けを行い、3未満の場合はランダムに割付ける。

（８）研究終了後の対応

　　本研究終了後は、この研究で得られた成果も含めて、研究責任医師は研究対象者に対し最も適切と考える医療を提供する。

# ５．観察および検査項目

（１）研究対象者背景

　　年齢、性別、現病歴、合併症、既往歴、喫煙歴、身長、体重

併用薬・併用治療（医療機器を含む）

（２）検査項目

＜スクリーニング期＞

血算（WBC、白血球像）、

生化学検査（AST、ALT、LDH、TP、Na、K、Cl、BUN、CRP）

その他の血液検査（プロカルシトニン、KL-6）

胸部X線、胸部CT、SpO_2_

＜介入期間＞および＜後観察期間＞

血算（WBC、白血球像）、

生化学検査（AST、ALT、LDH、TP、Na、K、Cl、BUN、クレアチニン、CRP）

ﾊﾞｲｵﾏｰｶｰ（日常診療で行われる採血の残余血清を用いる。Bio-Plex® マルチプレックスシステムを用いた網羅的サイトカイン解析、Hydropeoxides、TRX-1・HMGB-1、HSP70、S100A8/A9）

胸部X線、胸部CT、SpO_2_

（３）有害事象と副作用の確認

有害事象の内容、発現時期・消失時期、程度、処置、転帰、重篤性評価、テプレノン内服との関連性等をCRFに記載する。必要があれば自宅隔離を含めた隔離が解除になる時期まで追跡調査を行う。

（４）血清保存の取り扱い・輸送について

ﾊﾞｲｵﾏｰｶｰ検索目的で保存された血清は、本研究終了後、事務局から輸送の依頼があった後に保存された血清を下記に発送する。

〒700-8558 岡山県岡山市北区鹿田町2-5-1

岡山大学大学院医歯薬学総合研究科　　小児医科学　　津下充

電話　086-223-7151 （代表）

血清保存検体の輸送・取り扱いについては、国立感染症研究所　2019-nCoV (新型コロナウイルス)感染を疑う患者の検体採取・輸送マニュアル　に従う

https://www.niid.go.jp/niid/ja/diseases/ka/corona-virus/2019-ncov/2484-idsc/9325-manual.html

（頻回にupdateされるので最新版を参照のこと）。

| 項目／検査・調査時期 | 登録前7日以内  ＜スクリーニング期＞ | 登録時 | 介入期間 | | | | | 後観察期間 | | 気管内挿管時 |
| --- | --- | --- | --- | --- | --- | --- | --- | --- | --- | --- |
|  |  |  | Day1  テプレノン開始直前検査 | Day3 | Day5 | Day7 | Day10 | Day14 | Day21 |  |
| 適格性確認・登録 |  | ● |  |  |  |  |  |  |  |  |
| 確定診断 |  |  |  |  |  |  |  |  |  |  |
| COVID-19 PCR | ○ |  |  |  |  |  |  |  |  |  |
| 臨床検査 |  |  |  |  |  |  |  |  |  |  |
| 血算・生化学 | ○ |  | ● スクリーニング期の採血で代用可 |  | ▲ ±1日許容 |  | ▲ ±1日を許容 | ▲ | ▲ | ▲ |
| KL-6・ | △ |  |  |  |  |  |  |  |  |  |
| プロカルシトニン |  |  |  |  |  |  |  |  |  |  |
| 体温 | ○ | ● | ● 前日でも可 | ● | ● | ● | ● | ▲ | ▲ | ● |
| 投与酸素投与量 | 〇 |  | ● | ● | ● | ● | ● | ▲ | ▲ | ● |
| SpO2 | ○ |  | ● 前日でも可 | ● | ● | ● | ● | ▲ | ▲ | ● |
| ﾊﾞｲｵﾏｰｶｰ |  |  |  |  |  |  |  |  |  |  |
| ﾊﾞｲｵﾏｰｶｰ用  血清保存 |  |  | ▲ スクリーニング期の採血で代用可 |  | ▲ ±1日を許容 |  | ▲ ±1日を許容 |  |  |  |
| 効果判定 |  |  |  |  |  |  |  |  |  |  |
| 胸部X線 | △ |  |  | ▲ |  |  | ▲ |  | ▲ | ▲ |
| 胸腹部CT | △ |  |  |  |  |  | ▲ |  | ▲ | ▲ |
| 毒性評価 |  |  |  |  |  |  |  |  |  |  |
| 自・他覚症状 |  |  | ● 前日でも可 | ● | ● | ● | ● | ● | ▲ | ▲ |

**【スケジュール表】**

**早期退院・自宅安静となった症例については、**

**観察可能な期間内での観察とし、本研究のためだけの来院は不要とする**

△・▲必須ではないが可能な限り行うことを推奨する　　〇・△は同意取得前、●・▲は同意取得後に行う項目である。　＊血算・生化学・プロカルシトニン・KL-6・SpO2・胸部X線・胸部CTについては、同意前の情報を用いてもよい。

# ６．評価項目

（１）主要評価項目（Primary endpoint）

　　気管内挿管率

設定根拠：COVID-19肺炎 における重症化を示す明確な指標であるため。

気管内挿管を検討する目安：

１．十分な酸素療法下でPaO２が60Torr未満

２．換気量の低下（急性呼吸性アシドーシス）

３．意識レベルの低下、奇異呼吸、呼吸困難

（２）副次評価項目（Secondary endpoint）

　　①死亡率

　　②解熱までの期間

発熱：37.5℃以上　解熱：解熱剤の使用なく24時間以上37.5℃未満となること

　　③酸素投与量（入院時酸素投与量 [デバイスの形態は問わない]、

最大酸素投与量 [デバイスの形態は問わない]、酸素投与量の変化）

　　④胸部CTの画像変化の程度

⑤各血液データ値の絶対値および変化率

⑥重症度

⑦退院（隔離解除）できるまでの期間

⑧症状消失までの期間

　　⑨有害事象（CTCAE* version 5.0に基づく）*: Common Terminology Criteria for Adverse Events

　　設定根拠：①～⑧; テプレノンの有効性について比較検討するため。

　　　　　　　⑨; 患者の安全性を検討するため。

# ７．目標症例数および設定根拠

　　岡山大学病院では、35例（テプレノン投与群18例、非投与群17例）を予定している。

　　なお、研究全体では、参加施設数5施設、目標症例数100例（岡山大学病院; 35例、岡山医療センター；20例、その他各分担施設; 15例）を予定している。

【設定根拠】：

目的症例数100例は、現実的に集積できると考えられる数である。そもそも、COVID-19肺炎は新興感染症であるため、症例数の見込みが立ちにくい。研究参加施設の状況などを踏まえて設定した。

次に、この症例数において、群間で主要評価項目に有意な差があったときに、それを検証出来る確率（統計学的検出力）を算出する。非投与群の気管内挿管率を22%、投与群の気管内挿管率を10%と設定し、α=0.1（片側）としたとき、統計学的検出力は0.6406である。

なお、非投与群の気管内挿管率は国立感染症研究所を元にしている。Webに公開された情報によると、2020年3月9日時点でのCOVID-19感染症各例症例287例のうち、侵襲的換気について情報があるのは133症例であった。そのうち29症例（22%）で気管内挿管等が施行されたと報告されている（<https://bit.ly/3eiiMhg>）。

# ８．データの集計および統計解析方法

全記載（CRF記載、データベース作成、関連報告への記載等）は、研究責任医師、研究担当医師、または研究責任医師の許可を受けた研究協力者のいずれかが行う。研究責任医師は、データ及びその他の必要なすべての報告書について、正確性、完全性及び適時性を保証する。原資料（患者の医療記録など）に由来するCRFで報告されたデータは、その原資料と一致するものでなければならない。また、不一致が生じた場合には、文書にてその根拠を示し、研究責任医師が署名及び日付を記入したうえで、研究対象者のファイルに保管する。すべてのデータは原資料に由来するものである。

研究途中において、同意撤回により脱落した患者に関するデータは利用しない。その他の理由により脱落した患者のデータは、脱落前までの利用可能なデータを利用する。連続変数において極端な外れ値があったとしても、統計解析において除外しない。当初の統計解析計画（下記）の変更が必要と考えられる場合には、研究責任医師および研究分担医師が協議した上で統計解析計画の変更を行なう。また、中間解析は行なわない。

（１）解析対象集団

有効性の解析対象集団は、最小化法で割付けられ、同意撤回をしていない全症例と定義する。

安全性の解析対象集団は、割付治療を少なくとも1回以上施行した全症例とする。つまり、テプレノン投与群では1回以上のテプレノン投与がある患者、非投与群では試験参加期間中にテプレノン投与がない患者が該当する。

（２）解析方法

得られたデータは専用のデータベースに入力し、集計・解析を行う。

【主たる解析と判断基準】

全適格例の主要評価項目に関するデータがすべて収集されると見込まれる登録終了 1ヵ月後を目処に 行う。本試験の主要評価項目に関する主たる解析の目的は、テプレノン投与が十分な有効性と安全性を有するかどうかを評価することである。

主要評価項目である気管内挿管率について、帰無仮説である「非投与群の気管挿管率と投与群の気管挿管率が等しい」が棄却し、対立仮説が採択されれば有効と判断し、棄却されなければ臨床医学的には無効と判断する。試験全体の有意水準は片側 10%とする。多重性の調整は行わない。

【記述統計量】

CRFで収集され、データベースとして固定された変数について記述統計量を算出する。連続変数では、平均値、標準偏差、中央値、四分位範囲、第1四分位、第3四分位を示す。二値変数を含むカテゴリ変数では、頻度および割合を示す。

【有効性の解析 】

有効性の解析は、Intention-to-treat（ITT）の原則に従い、最小化法によって患者に割り付けられた治療に基づいて集計・解析する。ここでITT解析を、実際に行なわれた治療介入ではなく、治療介入を始める前に決定した投与群と非投与群の割付けに基づいて解析する方法と定義する。例えば、投与群に割付けられたが、何らかの理由により一度も投与することなく経過した患者も投与群として扱う。

主要評価項目（気管内挿管率）の解析

1. 主たる解析の方法

観察された気管内挿管率に基づいて「非投与群の気管挿管率と投与群の気管挿管率が等しい」という帰無仮説について二項検定を行う。対立仮説は「投与群の気管挿管率は非投与群の気管挿管率よりも小さい」とする。帰無仮説が棄却されれば有効と判断し、棄却されなければ臨床医学的に無効と判断する。

また、投与群の気管挿管リスクを非投与群の気管挿管リスクで割ることによってリスク比の算出を行なう。リスク比の区間推定には二項分布に基づく正確な90%信頼区間を用いる。

1. サブグループ解析の方法 以下に記す因子に基づくサブグループ解析を行う。

＜サブグループ解析を予定している因子＞

治療前因子：全適格例を対象に行う。

・70歳未満 / 70歳以上 ・ 男性 / 女性 ・ 喫煙歴有/無

副次評価項目（死亡率）の解析

主要評価項目の解析と同様に行なう。

副次評価項目（連続量）の解析

観察された変数について「非投与群の平均値と投与群の平均値が等しい」という帰無仮説についてWelch’s t 検定を行なう。対立仮説は「投与群の平均値は非投与群の平均値よりも少ない」とする。帰無仮説が棄却されれば有効と判断し、棄却されなければ臨床医学的に無効と判断する。Welch’s t 検定を採用する理由は、シミュレーション結果から正規性・等分散性の逸脱に対してStudent’s t検定やMann-Whitney 検定よりも頑健性を有していると評価されているためである。

また、投与群の平均値から非投与群の平均値で引くことによって、平均値差の算出を行なう。平均値差の区間推定にはt分布に基づく90%信頼区間を用いる。

サブグループ解析は、主要評価項目と同様に行なう。

この解析を行う副次評価項目は、解熱までの期間と酸素投与量である。

【安全性の解析】

投与群と非投与群のそれぞれについて、CTCAE version 5.0におけるGrade1からGrade5に該当する患者の人数および割合を算出する。

# ９．研究実施期間

　認定臨床研究審査委員会承認後のjRCT公表日から2024年12月31日

　（登録締切：2023年12月31日）

# １０．研究対象者の設定方針

（１）選択基準

　　以下の基準を全て満たすものを対象とする。

　　①同意取得日の年齢が20歳以上である

　　②PCRまたは抗原検査によりCOVID-19の診断が確定されている

　　③37.5度以上の発熱を認めている

　　④本研究の参加にあたり十分な説明を受けた後、十分な理解の上、患者本人の自由意思による文書同意が得られた患者（文書同意の取得方法については「12. インフォームド・コンセントを受ける手続き」を参照）

（２）除外基準

　　以下の基準のいずれかに該当するものは、本研究に組み入れないこととする。

1. 治療開始予定前2週間以内にテプレノン内服歴のある患者
2. 人工呼吸器管理あるいは体外式膜型人工肺（ECMO）管理を要する患者
3. SARS-CoV-2以外に明らかに活動性のある感染症を合併する患者（治療を要さないあるいは治療をしていても1か月以上進行のない非結核性抗酸菌症は登録可能）
4. 妊娠・授乳中の患者
5. 身元情報が得られない患者

　【設定根拠】

1. 安全性への配慮のため ②,③,④
2. 有効性評価への影響のため ①,③
3. 後日、連絡すべき新たな情報を伝えられない可能性が生じるため

⑤

# １１．研究の背景および科学的合理性の根拠（研究の合理性・妥当性）

研究の背景

既述（３．研究の背景、目的、意義）のように、COVID-19は世界的パンデミックの状況であり、抗ウイルス薬の開発とともに、重症化を予防する治療開発は喫緊の課題である。重症化予防の治療は、今後開発されていく抗ウイルス治療に併用して行われるべき治療戦略である。このため、有害事象ができるだけ少なく、治療薬へのアクセスが簡便な治療方法が理想的である。

研究の合理性・妥当性

本研究はテプレノンによるCOVID-19重症化予防効果の検討を目的としている。テプレノン投与群と非投与群のランダム化比較試験は本研究目的の達成に妥当な研究デザインと考える。

また発熱はCOVID-19の主要症状のひとつであり、解熱までの期間は主要評価項目として合理的である。

研究体制として岡山大学病院を含む岡山市内の公的5基幹病院とのネットワーク（岡山医療連携推進協議会）並びに中国四国地方の臨床試験ネットワーク、さらに関東と近畿の第二種感染症指定医療機関を予定し、予定症例数100例を登録する体制として適切な体制が整えられている。

COVID-19発生数の正確な予測は困難であるが、2020年4月3日時点で、国内で2600名を超える発症者を認め、発症者数は急激に増加傾向であることから研究期間内に予定登録数の登録は十分に可能と考えられる。

# １２．インフォームド・コンセントを受ける手続き

　研究責任医師または研究分担医師は、認定臨床研究審査委員会の承認が得られた説明文書を研究対象者本人に渡し、文書および口頭による十分な説明を行う。通常であれば、自由意思による同意を文書で得るが、本試験では感染防御の観点から、研究対象者本人から自由意思による口頭同意が得られた場合には、医師及び本試験に関わらない看護師等の立会人により同意書を作成する。研究対象者がCOVID-19感染症より回復した場合には、自宅隔離を含むすべての隔離規制が必要でなくなった時点で可能な限り再同意を得る。

研究対象者の同意に影響を及ぼすと考えられる有効性や安全性等の情報が得られたときや、研究対象者の同意に影響を及ぼすような実施計画等の変更が行われるときは、速やかに研究対象者に情報提供し、研究等に参加するか否かについて研究対象者の意思を予め確認するとともに、事前に認定臨床研究審査委員会の承認を得て説明文書・同意文書等の改訂を行い、研究対象者の再同意を得る。その際、研究対象者がCOVID-19感染症より回復していない場合の同意取得方法は、初回の同意取得方法と同一とする。

同意説明文書には、以下の内容を含むものとする。

①実施する特定臨床研究の名称、当該特定臨床研究の実施について実施医療機関の管理者の承認を得ている旨及び厚生労働大臣に実施計画を提出している旨

②実施医療機関の名称並びに研究責任医師の氏名及び職名（特定臨床研究を多施設共同研究として実施する場合にあっては、研究代表医師の氏名及び職名並びに他の実施医療機関の名称並びに当該実施医療機関の研究責任医師の氏名及び職名を含む。）

③特定臨床研究の対象者として選定された理由

④特定臨床研究の実施により予期される利益及び不利益

⑤特定臨床研究への参加を拒否することは任意である旨

⑥同意の撤回に関する事項

⑦特定臨床研究への参加を拒否すること又は同意を撤回することにより不利益な取扱いを受けない旨

⑧特定臨床研究に関する情報公開の方法

⑨特定臨床研究の対象者又はその代諾者の求めに応じて、研究計画書その他の特定臨床研究の実施に関する資料を入手又は閲覧できる旨及びその入手又は閲覧の方法

⑩特定臨床研究の対象者の個人情報の保護に関する事項

⑪試料等の保管及び廃棄の方法

⑫特定臨床研究に対する医薬品等製造販売業者等の関与の有無とその内容

⑬苦情及び問合せへの対応に関する体制

⑭特定臨床研究の実施に係る費用に関する事項

⑮他の治療法の有無及び内容並びに他の治療法により予期される利益及び不利益との比較

⑯特定臨床研究の実施による健康被害に対する補償及び医療の提供に関する事項

⑰特定臨床研究の審査意見業務を行う認定臨床研究審査委員会における審査事項その他当該特定臨床研究に係る認定臨床研究審査委員会に関する事項

⑱その他特定臨床研究の実施に関し必要な事項

# １３．個人情報等の取扱い（匿名化の方法を含む）

本研究に係わるすべての研究者は、「ヘルシンキ宣言」および「臨床研究法」を遵守して実施する。研究実施に係る情報を取扱う際は、研究対象者に研究独自の研究用IDを割り振り、氏名と研究用IDとの対応表を作成する。情報を研究事務局等に送付する場合は識別番号を使用し、研究対象者の氏名、生年月日などの情報が院外に漏れないよう十分配慮する。元データからは氏名を削除し、研究に用いる。研究期間を通して対応表ファイルはパスワードをかけ、漏洩しないように厳重に保管する。各機関における資料・試料及び個人情報についての管理責任者は、各機関の手順に従う。

研究の結果を公表する際は、氏名、生年月日などの直ちに研究対象者を特定できる情報を含まないようにする。また、研究の目的以外に、研究で得られた研究対象者の情報を使用しない。

# １４．研究対象者に生じる負担、予測されるリスク及び利益、これらの総合的評価並びに当該負担及びリスクを最小化する対策

（１）予想される利益

　　本研究に参加することによる直接的な利益はない。研究成果により将来の医療の進歩に貢献できる可能性がある。

（２）予想される不利益（副作用）

　研究対象者は10日間、テプレノンを内服する必要がある。被験治療により疾病を生じる可能性がある。テプレノン®の添付文書によれば、主な副作用は、AST/ALT上昇（0.1-5%未満）であり、その他便秘、下痢、嘔気、口渇、腹痛、腹部膨満感、頭痛、発疹、瘙痒感、総コレステロールの上昇、

眼瞼の発赤・熱感などがいずれも0.1%未満の確率とされている。

（３）有害事象発生時の研究対象者への対応

研究責任医師または研究分担医師は、有害事象を認めたときは、直ちに適切な処置を行うとともに、診療録ならびに症例報告書に記載する。また、被験薬の投与を中止した場合や、有害事象に対する治療が必要となった場合には、研究対象者にその旨を伝える。

（４）研究計画書等の変更

臨床研究を安全に実施する上で必要な情報を収集し、検討する。また、新たな安全性情報等が得られた場合、必要に応じて研究計画書および同意説明文書を変更する。研究計画書や同意説明文書の変更または改訂を行う場合は、あらかじめ、認定臨床研究審査委員会の承認を必要とする。実施計画の変更が生じた場合は、以下の届書を厚生労働大臣に提出する。

①実施計画事項変更届書（省令様式第二）

②変更後の実施計画

特定臨床研究の進捗に関する事項の変更については、変更後遅滞なく行う。実施計画の変更を行い、速やかに認定臨床研究審査委員会へ変更申請を行う。認定臨床研究審査委員会の承認が得られた後、以下の届書を厚生労働大臣に提出する。

①実施計画事項変更届書（省令様式第二）

②変更後の実施計画

実施計画について以下の軽微な変更を行った場合は、変更後10日以内に、変更内容を認定臨床研究審査委員会に通知する。また、届書（省令様式第三）を厚生労働大臣に提出する。

①特定臨床研究に従事する者の氏名の変更であって、特定臨床研究を従事する者の変更を伴わないもの

②地域の名称の変更又は地番の変更に伴う変更

（５）個々の研究対象者における中止基準

【研究中止時の対応】

研究責任医師または研究分担医師は、次に挙げる理由で個々の研究対象者について研究継続が不可能と判断した場合には、当該研究対象者についての研究を中止する。その際は、必要に応じて中止の理由を研究対象者に説明する。また、中止後の研究対象者の治療については、研究対象者の不利益とならないよう、誠意を持って対応する。

【中止基準】

①研究対象者から研究参加の辞退の申し出や同意の撤回があった場合

②COVID-19あるいはその合併症の進行により本研究の継続が困難であると判断される場合

③忍容不能な有害事象が発生した場合

④本研究全体が中止された場合

⑤その他の理由により、研究責任医師または研究分担医師が研究の中止が適当と判断した場合

　 （この場合、中止理由を記録しておく）

【試験治療中止時の対応】

研究責任医師または研究分担医師は、上記に挙げた理由で個々の研究対象者について被験治療継続ができないと判断した場合には、当該研究対象者についての試験治療を中止する。その際は、必要に応じて中止の理由を研究対象者に説明する。また、中止後の研究対象者の治療については、研究対象者の不利益とならないよう、誠意を持って対応する。

試験治療中止例のうち、被験薬投与を行った症例については、安全性評価のため観察を継続する。

# １５．情報の保管及び破棄の方法（保管期間を含む）

研究責任医師は、以下の事項に関する記録を作成する。

①本研究の対象者を特定する事項

②本研究の対象者に対する診療及び検査に関する事項

③本研究への参加に関する事項

④その他、本研究を実施するために必要な事項

本研究で収集した試料・情報は、研究終了後5年が経過した日までの間施錠可能な場所（各実施医療機関の定められた場所）で保存し、その後は個人情報に十分注意して廃棄する。保管する情報からは氏名、住所、生年月日などの直ちに個人を特定できる情報を削除して保管する。

研究責任医師は、研究等の実施に係わる必須文書（研究計画書、実施計画、本研究の対象者に対する説明及びその同意に係る文書、総括報告書その他の省令の規定により研究責任医師が作成した文書又はその写し、認定臨床研究審査委員会から受け取った審査意見業務に係る文書、モニタリングに関する文書、研究対象者識別コードリスト、同意書、症例報告書等の控、その他データの信頼性を保証するのに必要な書類または記録など）を、研究終了後5年が経過した日までの間施錠可能な場所（各実施医療機関の定められた場所）で保存し、その後は個人情報に十分注意して廃棄する。コンピュータ上にある情報は完全に削除し、紙媒体（資料）はシュレッダーにて裁断し廃棄する。

# １６．原資料等の閲覧に関する事項

本研究における原資料とは、以下のものをいう。

①研究者の同意および情報提供に関する記録

②診療記録、臨床検査データおよび画像検査フィルム等、症例登録時のデータ、および症例報告書の元となった記録

研究責任医師及び実施医療機関は、臨床研究に関連するモニタリング、監査、ならびに認定臨床研究審査委員会及び規制当局の調査の際に、原資料などのすべての臨床研究関連記録を直接閲覧に供する。

# １７．定期報告

研究代表医師は、本研究の実施状況に関する以下の内容について、原則として、実施計画書を厚生労働大臣に提出した日から起算して、1年ごとに、また、当該期間満了後2か月以内に認定臨床研究審査委員会に報告する。統一書式５定期報告書及び別紙様式３定期報告書を用いる。

①本研究に参加した研究対象者数

②本研究に係る疾病等の発生状況及びその後の経過

③本研究に係る省令又は研究計画書に対する不適合の発生状況及びその後の対応

④本研究の安全性及び科学的妥当性についての評価

⑤本研究に対する医薬品等製造販売業者等の関与に関する事項

また、認定臨床研究審査委員会が意見を述べた日から1か月以内に、以下の内容について別紙様式３を用いて厚生労働大臣に報告する。

①実施計画に記載されている認定臨床研究審査委員会の名称

②認定臨床研究審査委員会による本研究の継続の適否

③本研究に参加した対象者数

　研究代表医師は定期報告の内容及び委員会での承認・厚生労働大臣への提出に関して、研究責任医師に対して情報提供を行う。また、研究代表医師及び各研究責任医師は、各自の実施医療機関の管理者に対して、前述の定期報告書等を以って報告を行う。

# １８．研究の資金源、研究期間の研究に係る利益相反及び個人の収益等、研究等の研究に係る利益相反に関する状況

本研究は、岡山大学　学都基金　500万円で実施を予定する。

上記研究開発資金で実施するまでは、運営費交付金で実施する。

また、研究責任医師は「臨床研究法における利益相反管理ガイダンス」に従って、利益相反を申告し、その審査と承認を得るものとする。また、研究の利益相反と個人の利益相反に変更がないか、定期報告時に確認し、認定臨床研究審査委員会に報告する。

# １９．研究に関する情報公開の方法（研究計画の登録および研究結果の公表）

本研究は、臨床研究実施計画・研究概要公開システムJapan Registry of Clinical Trials(jRCT: https://jrct.niph.go.jp/) に登録する。また、本研究で得られた結果は、jRCT上、内科学会等の各関連学会等で発表し、専門学術誌で論文として公表する予定である。

# ２０．研究対象者等からの相談等への対応

研究対象者又はその代諾者等及びその関係者からの相談、問合せ、苦情等に対して、研究責任医師、研究分担医師は適切かつ迅速に対応する。

相談窓口責任者

岡山大学病院呼吸器・アレルギー内科　市原英基

　　住所：〒700-8558　岡山市北区鹿田町2-5-1

　　電話番号：086-235-7227（平日：９時～17時）

　　　　　　　086-235-6765（平日夜間、休日）

　　FAX番号：086-232-8227

# ２１．代諾者等からのインフォームド・コンセントを受ける場合の手順

本研究では、代諾者は設定しない。

# ２２．インフォームド・アセントを得る手続き（説明事項、説明方法含む）

　本研究では該当しない。

# ２３．緊急かつ明白な生命の危機が生じている状況での研究に関する要件の全てを満たしていることを確認するための手順

　本研究では該当しない。

# ２４．研究対象者等に経済的負担又は謝礼があればその内容

令和2年3月4日に発出された事務通知「新型コロナウイルス感染症に係る公費負担医療の取扱いについて」に基づき、本治療はCOVID-19陽性の患者を対象としており、公費負担医療で行われるため研究対象者への費用負担は発生しない。

また、本研究に参加することへの謝礼はない。

# ２５．疾病等および不具合が発生した場合の対応

（１）疾病等および不具合の定義

疾病等とは、特定臨床研究の実施に起因するものと疑われる疾病、障害もしくは死亡または感染症ならびにこれらを引き起こしうる不具合をいう。

（２）重篤な疾病等

　特定臨床研究の実施によると疑われる重篤な疾病等は、以下の通り定義する。

　１）死亡または死亡につながるおそれのあるもの

　２）１）以外の以下の疾病（その他の疾病）

①　治療のために医療機関への入院又は入院期間の延長が必要となるもの

②　障害

③　障害につながるおそれのある疾病等

④　①から③まで並びに死亡および死亡につながるおそれのある疾病等に準じて重篤であるもの

⑤　後世代における先天性の疾病又は異常

（３）研究との因果関係

全ての疾病と研究実施あるいは被験治療との関係性を、研究代表医師、研究責任医師又は分担医師が判断する。被験治療開始との時間的関係だけでなく、基礎疾患の経過、合併症、併用薬、研究手順、事故及びその他の外的因子などに起因することも考慮して判断する。

（４）予測性

疾病の予測性は、添付文書及びインタビューフォームに基づいて判断する。有害事象の性質、重症度または頻度が一致しない場合、未知の有害事象とする。予測される有害事象・不具合は、AST/ALT上昇（0.1-5%未満）、その他(便秘、下痢、嘔気、口渇、腹痛、腹部膨満感、頭痛、発疹、瘙痒感、総コレステロールの上昇、眼瞼の発赤・熱感など)がいずれも0.1%未満である。

（５）転帰

研究責任医師および研究分担医師（研究責任医師等）は、発現したすべての疾病に対して、適切な処置、治療を行い、症状が消失するまで又は検査値が基準値若しくは投与前値に復するまで、あるいは医学的に追跡調査の必要がないと治験責任医師等が判断するまで追跡調査を実施する。有害事象及び不具合の転帰を次のように分類する： 1)回復（症状、所見、臨検値の消失又は回復）、2)軽快（当該事象の軽減）、3)未回復（変化なし～悪化）、4)死亡、5)不明。

（６）研究責任医師等の責務（重篤な疾病の報告）

①研究責任医師等は、特定臨床研究の実施において重篤な疾病等の発生を知った場合には、研究対象者等への説明、治療等、必要な措置を講じなければならない。

②研究責任医師等は、疾病等の発生を知った時は、その旨を実施医療機関の管理者に報告した上で、厚生労働省令で定めるところにより、統一様式8を用いて、その旨を当該特定臨床研究の実施計画に記載されている認定臨床研究審査委員会に報告しなければならない。

③研究責任医師は、特定臨床研究の実施に起因すると疑われる疾病等の発生に関する事項で、厚生労働省令で定めるもの（以下表参照）を知ったときは、厚生労働省令で定めるところにより、別紙様式第2-1（医薬品）を用いて、その旨を厚生労働大臣に報告しなければならない。

| 予測性 | 疾病等 | 認定臨床研究審査  委員会への報告 | 厚生労働大臣への報告 |
| --- | --- | --- | --- |
| 予測できない | 死亡  死亡につながるおそれ | 7日以内 | 7日以内 |
|  | その他の疾病 | 15日以内 | 15日以内 |
| 予測できる | 死亡  死亡につながるおそれ | 15日以内 | 定期報告時 |
|  | その他の疾病 | 定期報告時 | 定期報告時 |

# ２６．健康被害に対する補償の有無及びその内容

本試験で用いられる薬剤は、通常の診療で一般的に広く使用されている薬剤である。その意味では日常診療を越えるリスクを患者に負わせることはなく、したがって、本臨床試験に参加することで生じた健康被害については、通常の診療と同様に病状に応じた適切な治療を保険診療として提供する。なお、本試験の対象は、COVID-19 陽性症例であり公費負担医療である（令和2年3月4日発出 「新型コロナウイルス感染症に係る公費負担医療の取扱いについて」）。

また、見舞金や 各種手当てなどの経済的な補償は行わない。

# ２７．不適合報告

研究責任医師は、当該臨床研究が法令等又は研究計画書に適合していない状態（「不適合」という。）であると知ったときは、速やかに、実施医療機関の管理者に報告しなければならない。研究分担医師は、不適合を知ったときは、速やかに研究責任医師に報告しなければならない。研究責任医師は、疾病等の発生を知ったとき速やかに、研究代表医師に通知しなければならに。また、研究代表医師はその旨を速やかに他の研究責任医師に情報提供する。この場合において、当該他の研究責任医師は、速やかに当該情報提供の内容を実施医療機関の管理者に報告する。研究代表医師は、不適合であって、特に重大なものが判明した場合においては、速やかに認定臨床研究審査委員会の意見を聴かなければならない。

「重大な不適合」とは、臨床研究の対象者の人権や安全性及び研究の進捗や結果の信頼性に影響を及ぼすものをいう。例えば、選択・除外基準や中止基準、併用禁止療法等の不遵守をいい、臨床研究の対象者の緊急の危険を回避するためその他医療上やむを得ない理由により研究計画書に従わなかったものについては含まない。研究責任医師は、重大な不適合が発生した場合は、再発防止策を講じ、研究分担医師や当該臨床研究に従事する者に周知するとともに、再発防止の徹底を図ることとする。委員会への定期報告時に、当該臨床研究に係る法令等又は研究計画書に対する不適合の発生状況及びその後の対応を報告しなければならない。

# ２８．研究の終了、中止

（１）研究の終了

　研究実施計画書に記載した主たる評価項目に係わるデータの収集を行うための期間が終了した時は、原則として１年以内に主要評価項目報告書を、臨床研究の内容に関する事項として記載した全ての評価項目に係るデータの収集を行うための期間が終了したときには原則として１年以内に総括報告書及びその概要をそれぞれ作成し､臨床研究審査委員会に提出する。

なお、主要評価項目報告書及び総括報告書を作成しなければならない時期が同時期になった場合は、総括報告書の作成により主要評価項目報告書の作成をしたものとする。

審査委員会から意見を聴いた主要評価項目報告書又は総括報告書及びその概要を実施医療機関の管理者に提出する。

また、委員会が意見を述べた日から１ヶ月以内に、主要評価項目報告書又は総括報告書の概要をjRCTに公表する。jRCTに公表された旨を管理者に報告する。

厚生労働大臣に総括報告書の概要を提出する際は、研究計画書、説明文書を併せて提出する。

（２）研究の中止

研究責任医師は、以下の事項に該当する場合は研究実施継続の可否を検討する。

１）本研究に使用するテプレノンの品質、安全性、有効性に関する重大な情報が得られたとき。

２）研究対象者のリクルートが困難で予定症例を達成することが困難であると判断されたとき。

３）予定症例数または予定期間に達する前に、試験の目的が達成されたとき。

４）認定臨床研究審査委員会により、実施計画等の変更の指示があり、これを受入れることが困難と判断されたとき。

認定臨床研究審査委員会により、中止の勧告あるいは指示があった場合は、研究を中止する。

研究の中止を決定した時は、その旨をその中止の日から10日以内に統一書式11を用いて、当該特定臨床研究の実施計画に記載されている認定臨床研究審査委員会に通知するとともに、様式第四を用いて、厚生労働大臣に届け出る。研究責任医師は、中止届を提出した場合であっても、臨床研究が終了するまでの間においては、疾病等報告、定期報告等を行う。また、特定臨床研究が終了するまで、特定臨床研究の進捗状況に関する事項の変更に該当する場合は、実施計画の変更の届出を行う。また、研究責任医師に研究中止の旨を情報提供する。各研究責任医師は、各機関の規定に従って対応する。

# ２９．研究対象者の健康、遺伝的特徴に関する重要な知見が得られる可能性がある場合の研究結果（偶発的所見を含む）の取扱い(研究結果の開示の方針、開示の方法等)

該当しない。

# ３０．研究に関する業務の一部を委託する場合には、当該業務内容及び委託先の監督方法

本研究に関する業務を他の機関に委託することはない。

# ３１．本研究で得られた試料・情報を将来の研究に用いる可能性

本研究で得られた情報を将来の研究に用いる可能性があるが、その場合は倫理委員会の承認を得ることとする。

# ３２．モニタリング及び監査の実施体制及び実施手順

（１）モニタリング

　本研究ではモニタリングを実施する。具体的な手順については、別途モニタリング計画書にて定める。

（２）監査

　本研究では実施しない。

# ３３．知的財産権、所有権の帰属先

この研究から成果が得られ、知的財産権などが生じる可能性がある。その取扱いについては、岡山大学で協議する。

# ３４．参考資料・文献リスト

1. Wang D, Hu B, Hu C, et al. Clinical Characteristics of 138 Hospitalized Patients with 2019 Novel Coronavirus-Infected Pneumonia in Wuhan, China. *JAMA - J Am Med Assoc*. 2020. doi:10.1001/jama.2020.1585

2. Hirakawa T, Rokutan K, Nikawa T, Kishi K. Geranylgeranylacetone induces heat shock proteins in cultured guinea pig gastric mucosal cells and rat gastric mucosa. *Gastroenterology*. 1996. doi:10.1053/gast.1996.v111.pm8690199

3. Ooie T, Takahashi N, Saikawa T, et al. Single oral dose of geranylgeranylacetone induces heat-shock protein 72 and renders protection against ischemia/reperfusion injury in rat heart. *Circulation*. 2001. doi:10.1161/hc3901.095771

4. Fujibayashi T, Hashimoto N, Jijiwa M, Hasegawa Y, Kojima T, Ishiguro N. Protective effect of geranylgeranylacetone, an inducer of heat shock protein 70, against drug-induced lung injury/fibrosis in an animal model. *BMC Pulm Med*. 2009. doi:10.1186/1471-2466-9-45

5. Kudoh S, Kato H, Nishiwaki Y, et al. Interstitial lung disease in Japanese patients with lung cancer: A cohort and nested case-control study. *Am J Respir Crit Care Med*. 2008. doi:10.1164/rccm.200710-1501OC

6. Namba T, Tanaka KI, Hoshino T, Azuma A, Mizushima T. Suppression of expression of heat shock protein 70 by gefitinib and its contribution to pulmonary fibrosis. *PLoS One*. 2011. doi:10.1371/journal.pone.0027296

7. Kim JS, Son Y, Jung MG, et al. Geranylgeranylacetone alleviates radiation-induced lung injury by inhibiting epithelial-to-mesenchymal transition signaling. *Mol Med Rep*. 2016. doi:10.3892/mmr.2016.5121
